# Differentiating between liver diseases by applying multiclass machine learning approaches to transcriptomics of liver tissue or blood-based samples

**DOI:** 10.1016/j.jhepr.2022.100560

**Published:** 2022-08-18

**Authors:** Stanislav Listopad, Christophe Magnan, Aliya Asghar, Andrew Stolz, John A. Tayek, Zhang-Xu Liu, Timothy R. Morgan, Trina M. Norden-Krichmar

**Affiliations:** 1 Department of Computer Science, University of California, Irvine, CA 92697, USA; 2 Medicine and Research Services, VA Long Beach Healthcare System, Long Beach, CA 90822, USA; 3 Division of Gastrointestinal & Liver Diseases, Department of Medicine, Keck School of Medicine, University of Southern California, Los Angeles, CA 90033, USA; 4 Division of General Internal Medicine, Harbor-UCLA Medical Center, University of California Los Angeles, Torrance, CA 90509, USA; 5 Department of Epidemiology and Biostatistics, University of California, Irvine, CA 92697, USA

**Keywords:** Classification, RNA sequencing, biomarker discovery, alcohol-associated liver disease, AC, alcohol-associated cirrhosis, AH, alcohol-associated hepatitis, AKR1B10, aldo-keto reductase family 1 member B10, BTM, blood transcription module, DE, differential expression, FPKM, fragments per kilobase of exon model per million reads mapped, GSEA, gene set-enrichment analysis, IG, information gain, IPA, Ingenuity Pathway Analysis, kNN, k-nearest neighbors, LR, logistic regression, LTCDS, liver tissue cell distribution system, LV, liver tissue, ML, machine learning, MMP, matrix metalloproteases, NAFLD, non-alcohol-associated fatty liver disease, PBMCs, peripheral blood mononuclear cells, RNA-seq, RNA sequencing, SCAHC, Southern California Alcoholic Hepatitis Consortium, SVM, support vector machine, TNF, tumor necrosis factor

## Abstract

**Background & Aims:**

Liver disease carries significant healthcare burden and frequently requires a combination of blood tests, imaging, and invasive liver biopsy to diagnose. Distinguishing between inflammatory liver diseases, which may have similar clinical presentations, is particularly challenging. In this study, we implemented a machine learning pipeline for the identification of diagnostic gene expression biomarkers across several alcohol-associated and non-alcohol-associated liver diseases, using either liver tissue or blood-based samples.

**Methods:**

We collected peripheral blood mononuclear cells (PBMCs) and liver tissue samples from participants with alcohol-associated hepatitis (AH), alcohol-associated cirrhosis (AC), non-alcohol-associated fatty liver disease, chronic HCV infection, and healthy controls. We performed RNA sequencing (RNA-seq) on 137 PBMC samples and 67 liver tissue samples. Using gene expression data, we implemented a machine learning feature selection and classification pipeline to identify diagnostic biomarkers which distinguish between the liver disease groups. The liver tissue results were validated using a public independent RNA-seq dataset. The biomarkers were computationally validated for biological relevance using pathway analysis tools.

**Results:**

Utilizing liver tissue RNA-seq data, we distinguished between AH, AC, and healthy conditions with overall accuracies of 90% in our dataset, and 82% in the independent dataset, with 33 genes. Distinguishing 4 liver conditions and healthy controls yielded 91% overall accuracy in our liver tissue dataset with 39 genes, and 75% overall accuracy in our PBMC dataset with 75 genes.

**Conclusions:**

Our machine learning pipeline was effective at identifying a small set of diagnostic gene biomarkers and classifying several liver diseases using RNA-seq data from liver tissue and PBMCs. The methodologies implemented and genes identified in this study may facilitate future efforts toward a liquid biopsy diagnostic for liver diseases.

**Lay summary:**

Distinguishing between inflammatory liver diseases without multiple tests can be challenging due to their clinically similar characteristics. To lay the groundwork for the development of a non-invasive blood-based diagnostic across a range of liver diseases, we compared samples from participants with alcohol-associated hepatitis, alcohol-associated cirrhosis, chronic hepatitis C infection, and non-alcohol-associated fatty liver disease. We used a machine learning computational approach to demonstrate that gene expression data generated from either liver tissue or blood samples can be used to discover a small set of gene biomarkers for effective diagnosis of these liver diseases.

## Highlights

• Diagnosis of liver diseases involves multiple tests, often including invasive liver biopsy.
• Machine learning computational methods were used with gene expression data.
• These methods accurately classified alcohol-associated and non-alcohol-associated liver diseases.
• Promising blood-based diagnostic gene biomarkers were identified.
• These methods and biomarkers could aid in the development of liquid biopsy for liver disease.

## Graphical abstract

**Figure:**
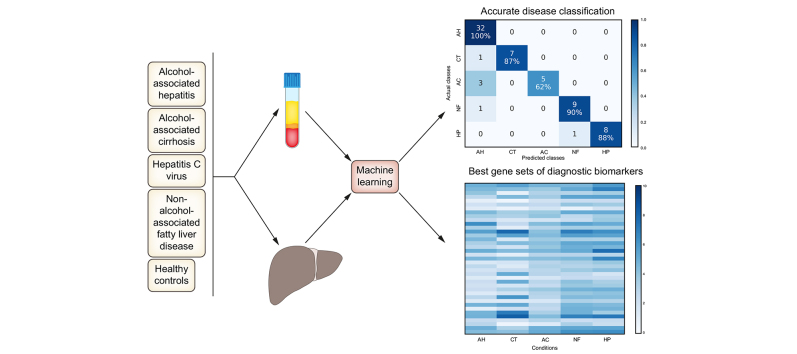

## Introduction

Liver disease is responsible for 2 million deaths worldwide annually, ranking as one of the leading causes of death in the world.^1^ Alcohol-associated hepatitis (AH) is one of the deadliest liver diseases.^2^ Other liver disorders such as alcohol-associated cirrhosis (AC), chronic HCV infection, and non-alcohol-associated fatty liver disease (NAFLD) are less deadly but are more widespread. Distinguishing between various alcohol-associated and non-alcohol-associated liver diseases typically requires multiple lab tests that often culminate in liver biopsy.^3^ The diagnosis is further complicated because factors that promote liver disease, such as viral hepatitis, obesity, and alcohol misuse, may overlap. Distinguishing AH and AC may be especially difficult and is thus an area of unmet clinical need. Presently, liver biopsy is regarded as the gold standard for confirming liver disease diagnosis and staging fibrosis severity. This approach has several limitations, such as procedural risk of internal bleeding, high cost, and patient dissatisfaction. While various clinical parameters, blood panels, and imaging tests have been used to supplement liver biopsy, they are not sufficiently effective to fully replace liver biopsy.^4^ Development of a liquid biopsy that is as accurate as liver biopsy for diagnosis of liver disease would improve quality of patient care and reduce healthcare costs. This process relies on identifying effective blood-based diagnostic biomarkers.

Development of liquid biopsies using blood-based biomarkers holds great promise when used with genomic data. For example, one recent study on epigenetic universal cancer biomarkers utilized DNA methylation markers.^5^ While the field is expanding, many of the clinically used blood-based biomarkers are cancer-specific.^6^ There is a shortage of effective diagnostic blood-based biomarkers for liver diseases. Presently many of the established biomarkers for liver disease are proteins found in blood serum such as albumin.^7^ Circulating microRNAs such as miR-122 and miR-155 have also been identified as diagnostic biomarkers for a range of liver diseases.^7^ Several previous studies have established that gene expression profiling of peripheral blood mononuclear cells (PBMCs) can be used to characterize HBV, HCV, and primary biliary cholangitis.[8], [9], [10], [11] Serum markers have been used to distinguish between alcohol-associated and non-alcohol-associated liver diseases using several machine learning (ML) models.^12^ Liver tissue gene expression in combination with clinical parameters has been used to establish prognosis in patients with AH and HCV-related early-stage cirrhosis.^13^^,^^14^

In this study, we chose to analyze gene expression in PBMCs for a variety of reasons. PBMCs can be extracted from a blood sample, pelleted and flash frozen, and provide ample material for RNA sequencing (RNA-seq). The differences in gene expression of PBMCs have been shown to reflect disease state. Additionally, we also characterized gene expression of liver tissue. The liver tissue served as a benchmark against which PBMCs could be compared, since pathology of liver tissue is currently the standard for distinguishing between liver diseases.

We were primarily interested in distinguishing between AH and AC, which may have similar clinical presentations. To establish the robustness of our models in discriminating between inflammatory liver diseases, we further sought to distinguish alcohol-associated liver diseases from non-alcohol-associated liver diseases, such as NAFLD and HCV. Therefore, we have trained ML models to differentiate between these liver diseases and healthy controls. As part of the classification process, we have also identified effective diagnostic gene biomarkers.

Like most individual biomedical research studies, ours was limited to a small number of participant samples due to the high costs of recruitment, sequencing, data storage, and data analysis. The gene expression data is also inherently highly dimensional. Datasets that contain more features than samples are difficult to classify. Therefore, it was crucial in our study to use statistical and ML techniques tailored for handling small sample and large feature sizes. In addition to identifying useful PBMC-based diagnostic biomarkers of liver diseases, our secondary goal was to evaluate multiple bioinformatic pipelines in the context of analyzing small sample size RNA-seq data. Special focus was given to feature selection, wherein, we compared several different feature selection approaches. Overall, our ML pipeline demonstrated excellent classification performance across the liver diseases using both liver tissue and PBMCs.

## Materials and methods

### Study population

This study was primarily conducted using biospecimens collected from participants enrolled by the Southern California Alcoholic Hepatitis Consortium (SCAHC). The protocol was approved by the IRB, and informed written consent was obtained from all participants. The liver tissue from participants with AC, NAFLD, HCV, and healthy controls were obtained from the liver tissue cell distribution system (LTCDS) at University of Minnesota. Participant demographics are outlined in Table 1, Table 2. We summarized the age, MELD (model for end-stage liver disease) score, Maddrey's discriminant function, BMI, sex, and ethnicity of our study population. As expected, the NAFLD group had the highest mean BMI, while the AH group had the highest mean MELD and Maddrey's discriminant function scores.

**Table 1:** Study population demographics (PBMCs).

	PBMC samples				
	AH	CT	AC	NF	HP
	(n = 38)	(n = 20)	(n = 40)	(n = 20)	(n = 19)
Age, mean ± SD	47.3 ± 11.5	35.9 ± 15.6	54.5 ± 9.7	52.2 ± 14.9	58.9 ± 7.4
MELD, mean ± SD	25 ± 3.8	7.3 ± 2.6	13.4 ± 5.8	8.9 ± 4	8.9 ± 2.8
Maddrey’s DF, mean ± SD	52.6 ± 20.7	2.4 ± 8.1	21.1 ± 19.1	7.7 ± 14.1	6.7 ± 7.1
BMI, mean ± SD	30 ± 6.2	27 ± 3.5	30.4 ± 5.1	36.5 ± 6	29.6 ± 5.9
Sex, n (%)					
Female	1 (2.6%)	8 (40.0%)	0 (0.0%)	4 (20.0%)	8 (42.1%)
Male	37 (97.4%)	12 (60.0%)	40(100.0%)	16 (80.0%)	11 (57.9%)
Ethnicity, n (%)					
Hispanic	25 (65.8%)	8 (40.0%)	25 (62.5%)	9 (45.0%)	10 (52.6%)
NHW	10 (26.3%)	0 (0.0%)	13 (32.5%)	7 (35.0%)	4 (21.1%)
Black	2 (5.3%)	2 (10.0%)	1 (2.5%)	2 (10.0%)	5 (26.3%)
Other	1 (2.6%)	10 (50.0%)	1 (2.5%)	2 (10.0%)	0 (0.0%)
Source	SCAHC	SCAHC	SCAHC	SCAHC	SCAHC

AC, alcohol-associated cirrhosis; AH, alcohol-associated hepatitis; CT, healthy controls; DF, discriminant function; HP, HCV infection; MELD, model for end-stage liver disease; NF, non-alcoholic fatty liver disease; NHW, non-Hispanic White; SCAHC, Southern California Alcoholic Hepatitis Consortium.

**Table 2:** Study population demographics (Liver).

	Liver tissue samples				
	AH	CT	AC	NF	HP
	(n = 32)	(n = 8)	(n = 8)	(n = 10)	(n = 9)
Age, mean ± SD	43.3 ± 11.3	55.4 ± 4.3∗	54.2 ± 6.9∗	56.8 ± 11.6	56.8 ± 7.6
MELD, mean ± SD	25.1 ± 5.7	NA	NA	28 ± 5.9∗	27.2 ± 7.5∗
Maddrey’s DF, mean ± SD	52.3 ± 22.1	NA	NA	NA	NA
BMI, mean ± SD	29.4 ± 5.9	NA	NA	NA	NA
Sex, n (%)					
Female	3 (9.4%)	0 (0.0%)	0 (0.0%)	0 (0.0%)	0 (0.0%)
Male	29 (90.6%)	7 (87.5%)	5 (62.5%)	10 (100.0%)	9 (100.0%)
Ethnicity, n (%)					
Hispanic	25 (78.1%)	NA	0 (0.0%)	0 (0.0%)	1 (11.1%)
NHW	5 (15.6%)	NA	4 (50.0%)	7 (70.0%)	5 (55.5%)
Black	1 (3.1%)	NA	0 (0.0%)	1 (10.0%)	2 (22.2%)
Other	1 (3.1%)	NA	0 (0.0%)	0 (0.0%)	0 (0.0%)
Source	SCAHC	LTCDS	LTCDS	LTCDS	LTCDS

The ethnicity and sex percentages may not add up to 100% due to missing data.

AC, alcohol-associated cirrhosis; AH, alcohol-associated hepatitis; CT, healthy controls; DF, discriminant function; HP, HCV infection; LTCDS, liver tissue cell distribution system; MELD, model for end-stage liver disease; NF, non-alcoholic fatty liver disease; NHW, non-Hispanic White; SCAHC, Southern California Alcoholic Hepatitis Consortium.

∗ Missing age for 3 AC participants, MELD for 2 NF participants, and MELD for 4 HP participants.

The biospecimens consisted of 137 PBMC samples and 67 liver tissue (LV) samples. The liver diseases represented were encoded with 2 letter symbols (as presented in the tables and figures) as follows: alcohol-associated hepatitis (AH), alcohol-associated cirrhosis (AC), NAFLD (NF), chronic HCV (HP), and healthy controls (CT). All PBMC and liver tissue samples were collected from distinct participants except for 19 participants with AH that provided both sample types. Most of the AC participants within the SCAHC study were expected to be in-patients with decompensated cirrhosis. The inclusion and exclusion criteria can be found in the supplementary materials. Best efforts were made during recruitment of the AH and non-AH groups within the SCAHC study to match based on age, sex, and ethnicity. Severity-based matching was not possible due to small sample size.

### Sample collection

The blood samples and liver biopsies from participants with AH were collected before starting treatment. Blood samples from all other groups were collected at entry into the study. PBMCs were freshly isolated from the blood samples by Ficoll-Histopaque (GE Healthcare) gradient centrifugation, flash frozen, and then stored in a liquid nitrogen tank. The AH biopsy sample was placed in a cryovial containing *RNAlater* (Invitrogen) and flash frozen in liquid nitrogen. The liver tissue samples for healthy controls, AC, NALFD, and HCV conditions were obtained from University of Minnesota LTCDS.

### Sample data preprocessing

#### RNA sequencing and alignment

Several samples were removed prior to use in our study, due to poor read quality.^15^ The trimmed, filtered, and decontaminated reads were aligned to the hg38 (GRCh38 assembly) human reference genome using STAR 2.6.0^16^ with default settings (STARCQ), and annotated with Ensembl release 91 (Dec 2017).

#### Partitioning samples into 4 data sets

We divided our data into 4 datasets, which we refer to as follows: LV 2-Way, LV 3-Way, LV 5-Way, and PBMC 5-Way. LV 2-Way included liver tissue samples from participants with AH (n = 32) and healthy (n = 8) conditions. The LV 3-Way included liver tissue from participants with AH (n = 32), healthy (n = 8), and AC (n = 8) conditions. The LV 5-Way included liver tissue from participants with AH (n = 32), healthy (n = 8), AC (n = 8), NAFLD (n = 10), and HCV (n = 9) conditions. The PBMC 5-Way included PBMC samples from participants with AH (n = 38), healthy (n = 20), AC (n = 40), NAFLD (n = 20), and HCV (n = 19) conditions.

#### Validation dataset

We validated our liver tissue ML models using the GSE142530 dataset.^17^ This dataset contained liver tissue RNA-seq data from participants with AH (n = 10), healthy (n = 12), and AC (n = 6) conditions. We utilized the counts data that had been generated with DESeq2 and deposited in GEO.^18^ Publicly available RNA-seq gene expression data from PBMCs was not available for the conditions in our study, and therefore, only the liver tissue datasets were validated using independent data.

#### Analysis of gene expression data

For each sample and workflow within our data, standard fragments per kilobase of exon model per million reads mapped (FPKM) values were directly extracted from the corresponding alignment results (BAM files) using the Cuffquant utility of the Cufflinks suite (release 2.2.1).^19^ The FPKM counts were then further normalized using Cuffdiff geometric normalization. The RNA-seq counts were transformed using ln(1+count) formula. This transformation greatly reduced count variance and improved classification accuracies (Fig. S1 and Fig. S2). The validation dataset counts generated by DESeq2 were presumably normalized using DESeq2’s default median of ratios method, which is equivalent to Cuffdiff’s geometric normalization. These counts were also transformed using ln(1+count) formula.

### Classification and feature selection architecture

#### Overview of classification and feature selection pipeline

**Fig. 1:**
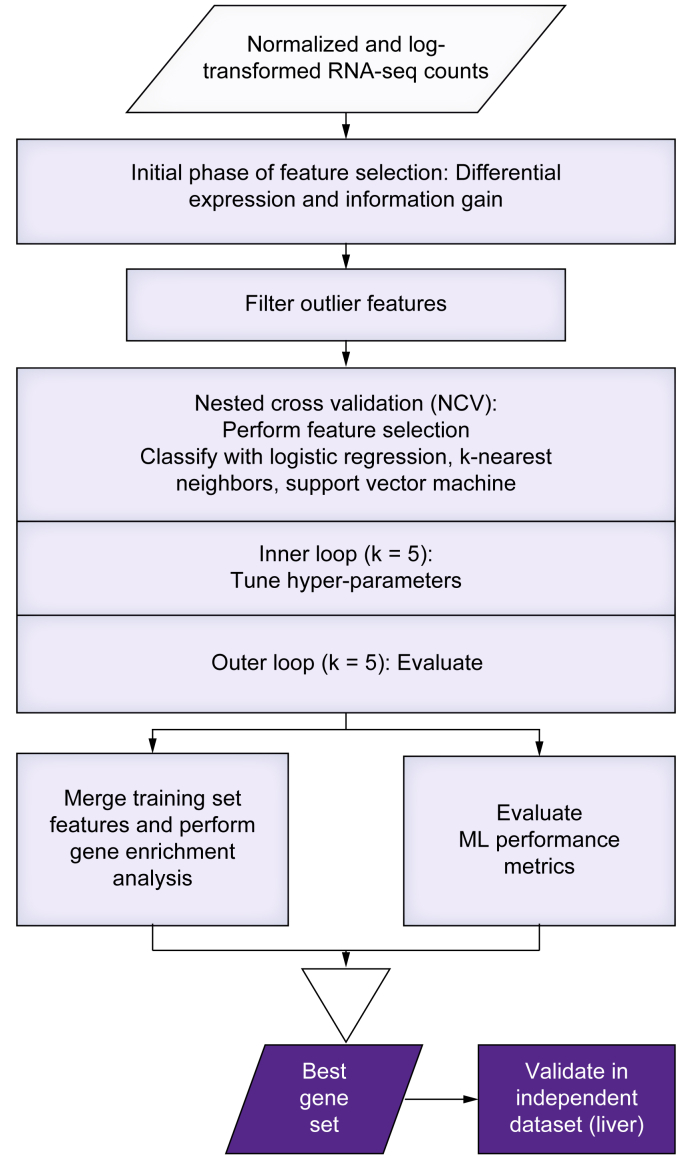
Diagram outlining the flow of processes in the machine learning feature selection and classification pipeline. ML, machine learning; RNA-seq, RNA sequencing.

The classification and feature selection pipeline process flow is visualized in Fig. 1. Feature selection was performed on each training set using differential expression (DE) and information gain (IG) methods. The DE and IG feature selection methods are referred to as filter feature selection methods.^20^ DE feature selection was performed using Cuffdiff, while the IG feature selection was implemented using scikit-learn (version 0.23.2+) package’s implementation of IG algorithm.^21^

Regardless of the feature selection method used, once the features were selected, the classification process was similar. The classifiers were evaluated using k-fold nested cross-validation (k outer and inner = 5). The feature selection was performed inside of inner and outer loops of nested cross-validation. The classification performance was primarily evaluated using confusion matrices, overall, and per-class accuracies. The features selected in the outer loop of nested cross-validation were merged together to form the candidate gene set, if they appeared in at least 4 out of 5 training sets. The resulting candidate gene sets were then evaluated using gene enrichment analysis. A combination of feature size, overall accuracy, per-class accuracies, and gene enrichment analysis were then used to pick a best gene set for each dataset. In the case of liver tissue datasets, the best gene sets were then further evaluated in an independent validation dataset. We used Python 3.7+ for all ML analysis, and all of the classifiers were implemented in scikit-learn package. The power size calculation was performed in R.

#### ML classifiers

The ML analysis for all 4 of our datasets was performed and was reported in this study using logistic regression (LR), k-nearest neighbors (kNN), and support vector machine (SVM) classifiers. The corresponding hyper-parameters used during grid search can be found in the codebase.

### In silico biological validation and best gene selection

The genes selected during feature selection were computationally evaluated for biological relevance using gene enrichment analysis via Enrichr with pathway, tissue, and disease Enrichr libraries.^22^ The resulting hits were filtered using an adjusted *p* value cut-off of 0.05 and regular expression matching. The terms used for pathways regular expression matching included names of various immune system pathways. The terms used for tissue regular expression matching included names of various cell types that comprise blood and liver tissues. The terms used for disease regular expression matching included the conditions within this study (AH, AC, NAFLD, HCV) along with a few other liver and blood disorders.

To compare the *in silico* biological relevance of many different gene sets, we devised a simple tallying system to count the number of hits within pathway, tissue, and disease libraries that passed the adjusted *p* value cut-off and regular expression matching. For each of the 4 datasets, we identified a gene set (Box 1) that exhibited both high classification accuracy and highly relevant *in silico* biological validation results using Enrichr. We have also provided the fold changes of the best genes for Liver 5-Way and PBMC 5-Way datasets (Tables S6 and S7).

Box 1 **Best gene sets for Liver 2-Way, Liver 3-Way, Liver 5-Way, and PBMC 5-Way datasets.**

**Figure:**
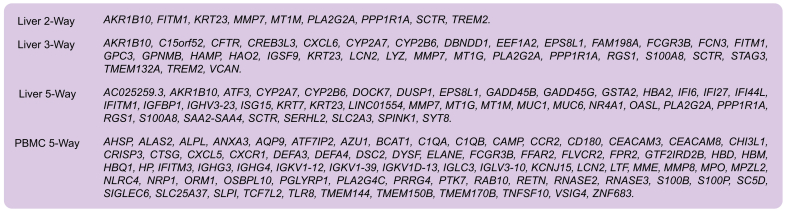

Additionally, we evaluated the best gene sets for Liver 5-Way and PBMC 5-Way datasets using Ingenuity Pathway Analysis (IPA), gene set-enrichment analysis (GSEAPreranked), and blood transcription module (BTM) analysis (BloodGen3Module) tools.[23], [24], [25] Blood transcription module analysis was performed with the PBMC 5-Way dataset only, since this method is specific to blood-based samples. Notably, this technique was recently utilized to analyze RNA-seq data from PBMCs to predict response to corticosteroid therapy in patients with AH.^26^ Since these tools utilize different knowledgebases and statistical methods, they provided complementary pathway annotations. The methods and results for these tools are provided in the supplementary information.

### Independent test dataset validation

After the best gene set was selected for each of our 3 liver tissue datasets, the independent validation dataset was utilized as follows. The ML classifier that performed best with the selected gene set was trained on the entirety of the corresponding liver dataset (*i.e.*, LV 2-Way, LV 3-Way, or LV 5-Way), using only the best genes selected for that dataset. The hyper-parameters for this classifier were selected by performing a regular cross-validation over the entirety of the corresponding liver dataset. The trained model was then tested in the independent dataset. While the PBMC 5-Way model could not be tested in an independent dataset set due to lack of appropriate public data, the methods prior to the independent dataset evaluation were the same for both liver and PBMC tissues. Therefore, we are confident that the PBMC genes identified in this study will have reasonable generalization. Additionally, the PBMC dataset had twice as many samples available for training and testing as the liver dataset, thereby also strengthening confidence in the best PBMC gene set. For additional details regarding methods, please refer to the supplementary methods and CTAT table.

## Results

### Classification of LV 2-Way (AH vs. Healthy)

**Fig. 2:**
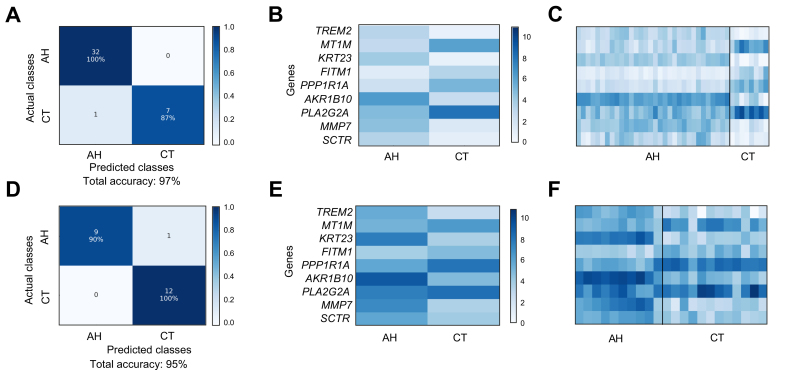
Confusion matrices and RNA-seq count heatmaps corresponding to the best gene set of LV 2-Way dataset. (A) Confusion matrix for classification of LV 2-Way dataset using best gene set. The diagonal contains the number and percentage of the correctly predicted samples. (B) Heatmap of best LV 2-Way gene set averaged per condition. (C) Per replicate heatmap of best LV 2-Way gene set. (D) Confusion matrix for classification of AH and CT samples within validation dataset. (E) Heatmap of best gene set within validation dataset averaged per condition. (F) Per replicate heatmap of best gene set within validation dataset. AH, alcohol-associated hepatitis; CT, healthy controls; LV, liver tissue; RNA-seq, RNA sequencing.

We developed many of our approaches described in the Methods section while first analyzing the binary dataset of AH *vs.* healthy samples. The task of distinguishing between AH and healthy samples proved simple, with accuracy as high as 100% depending on feature size, classifier, and feature selection methods. Based on their classification performance and runtime in the LV 2-Way dataset we chose to use LR, kNN, and SVM classifiers for the remaining datasets. The gene sets produced via various feature selection and outlier filtering strategies were also computationally evaluated for biological relevancy using Enrichr (Table S18). We selected the best gene set for our LV 2-Way dataset and then validated it in the independent test dataset. Using the best gene set of only 9 genes, we attained 97% classification accuracy within the LV 2-Way dataset, and 95% accuracy in the validation dataset, as visualized using confusion matrices (Fig. 2). Heatmaps of the RNA-seq counts per condition as an average and for each replicate show that the 2 conditions are very distinct from each other in both our LV 2-Way dataset and the independent dataset (Fig. 2). The best gene set for each of the 4 datasets is shown in Box 1.

### Classification of LV 3-Way (AH vs. Healthy vs. AC)

**Fig. 3:**
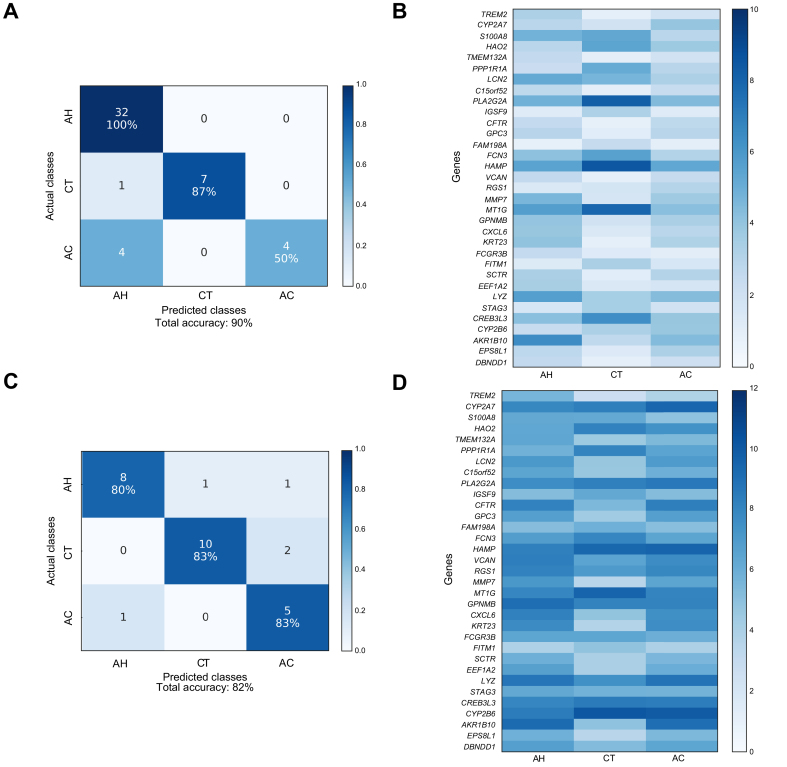
Confusion matrices and RNA-seq count heatmap corresponding to the best gene set of LV 3-Way dataset. (A) Confusion matrix for classification of LV 3-Way dataset using best gene set identified by filter feature selection. (B) RNA-seq count heatmap of best LV 3-Way gene set averaged per condition. (C) Confusion matrix for classification of AH, AC, and CT samples within independent validation dataset. (D) RNA-seq count heatmap of best gene set within independent validation dataset (AH, AC, and CT) averaged per condition. AC, alcohol-associated cirrhosis; AH, alcohol-associated hepatitis; CT, healthy controls; LV, liver tissue; RNA-seq, RNA sequencing.

Having successfully distinguished between AH and healthy samples with high accuracy, we proceeded to the more difficult multiclass classification task of discriminating between multiple liver diseases at once. Our classifiers peaked around 90% overall accuracy within our LV 3-Way dataset (Table S19). We identified the best gene set by examining the accuracies and *in silico* biological validation scores of each gene set produced by various feature selection configurations (Table S19 and S20). The top Enrichr hits for the LV 3-Way dataset are shown in Table S21. Using the best gene set comprised of 33 genes, we attained 90% overall accuracy in the LV 3-Way dataset (via nested cross-validation) and 82% overall accuracy in the independent validation dataset. The confusion matrices and the heatmaps of RNA-seq counts corresponding to the best gene set within LV 3-Way and the independent validation datasets are displayed in Fig. 3.

### Classification of LV 5-Way (AH vs. Healthy vs. AC vs. NAFLD vs. HCV)

**Fig. 4:**
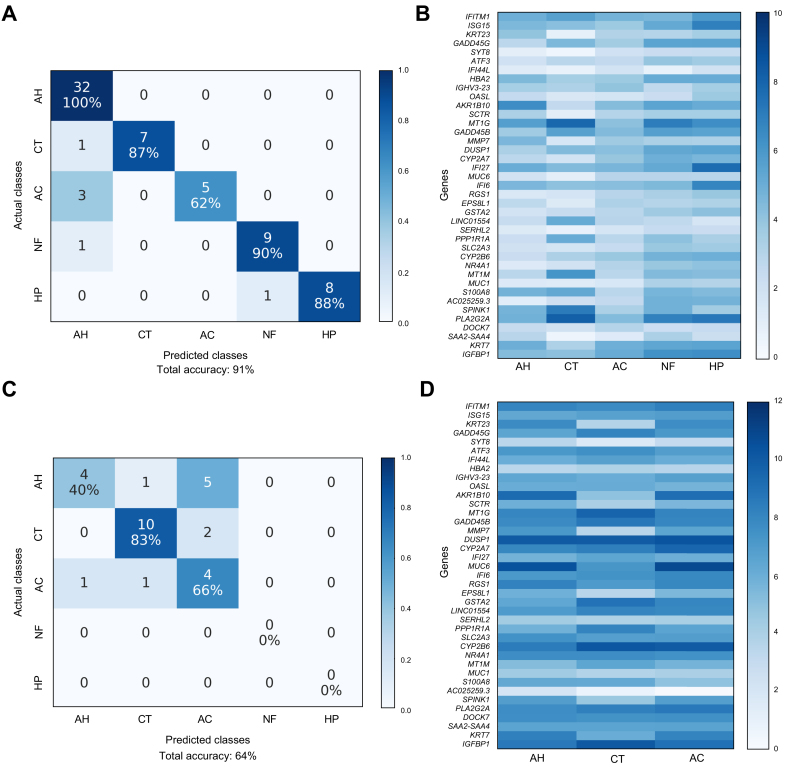
Confusion matrices and RNA-seq count heatmaps corresponding to the best gene set of LV 5-Way dataset. (A) Confusion matrix for classification of LV 5-Way dataset using best gene set identified by filter feature selection. (B) RNA-seq count heatmap of best LV 5-Way gene set averaged per condition. (C) Confusion matrix for classification of AH, AC, and CT samples within independent validation dataset. (D) RNA-seq count heatmap of best gene set within independent validation dataset (AH, AC, and CT) averaged per condition. AC, alcohol-associated cirrhosis; AH, alcohol-associated hepatitis; CT, healthy controls; HP, chronic HCV infection; LV, liver tissue; NF, non-alcohol-associated fatty liver disease; RNA-seq, RNA sequencing.

The LV 5-Way dataset was the most complex liver tissue dataset in the study. While AH and healthy groups were generally classified with high accuracy, the remaining conditions proved to be more challenging to appropriately classify (Fig. 4). The classifiers peaked at around 90% overall accuracy within the LV 5-Way dataset (Table S22). We identified the best gene set using a combination of classification performance and *in silico* biological validation metrics (Tables S22 and S23). For the annotations of the best gene set for LV 5-Way, the top hits using Enrichr are shown in Table S24, IPA in Table S28, and GSEA in Table S30. Using the best gene set comprised of 39 genes, we attained 91% overall accuracy within the LV 5-Way dataset (via nested cross-validation) and 64% overall accuracy in the validation dataset. While the overall classification accuracy in the independent dataset was lower than in the LV 3-Way testing, this was expected since the LV 5-Way gene set was based on 2 additional liver diseases (NAFLD and HCV), which were not present in the independent dataset. Notably, there were no samples from the independent dataset that were misclassified as NAFLD or HCV. The confusion matrix and the heatmap of RNA-seq counts corresponding to the best gene set within LV 5-Way and the independent validation datasets are shown in Fig. 4.

### Classification of PBMC 5-Way (AH vs. Healthy vs. AC vs. NAFLD vs. HCV)

**Fig. 5:**
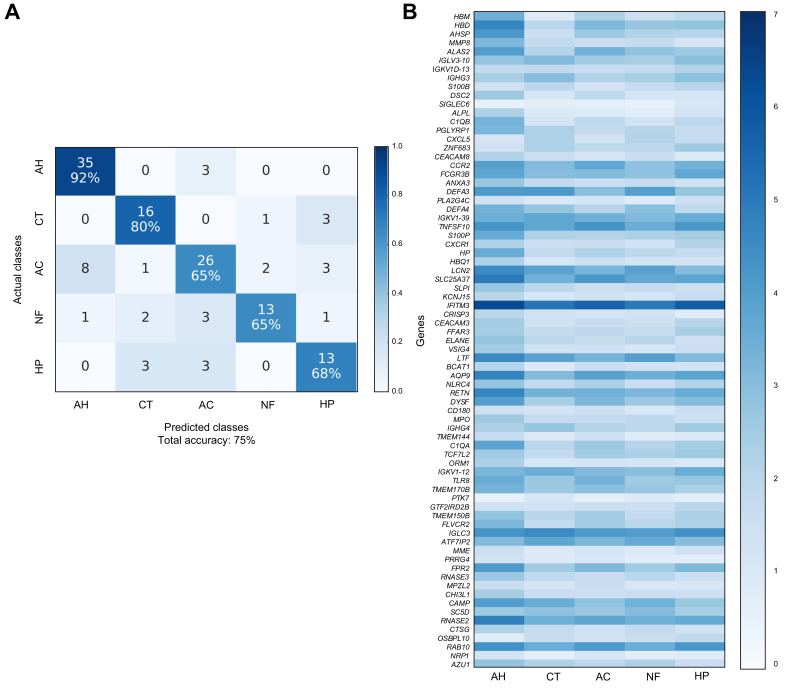
Confusion matrices and RNA-seq count heatmaps corresponding to the best gene set of PBMC 5-Way dataset. (A) Confusion matrix for classification of PBMC 5-Way dataset using best gene set identified by filter feature selection. (B) RNA-seq count heatmap of best PBMC 5-Way gene set averaged per condition. AC, alcohol-associated cirrhosis; AH, alcohol-associated hepatitis; CT, healthy controls; HP, chronic HCV infection; NF, non-alcohol-associated fatty liver disease; PBMC, peripheral blood mononuclear cells; RNA-seq, RNA sequencing.

Having achieved high classification accuracies in liver datasets, we broadened the scope of our study by applying these same ML models and strategies to our PBMC dataset. The classifiers tested peaked at 75% overall accuracy (Table S25). We identified the best gene set using a combination of classification performance and *in silico* biological validation metrics (Tables S25 and S26). For the annotations of the best gene set for PBMC 5-Way, the top hits using Enrichr are shown in Table S27, IPA in Table S29, GSEA in Table S31, and BloodGen3Module in Table S32. Using the best gene set comprised of 75 genes, we attained 75% overall accuracy in PBMC 5-Way dataset (via nested cross-validation). Because we could not obtain public RNA-seq data from PBMCs for several of our liver diseases, we could not validate the PBMC genes and classification performance in an independent data set. However, since the methods used to identify the best gene set were identical for both liver and PBMC datasets, we are confident of our results. The confusion matrix and the heatmap of RNA-seq counts corresponding to this gene set are shown in Fig. 5.

## Discussion

To the best of our knowledge, this is the first study to utilize ML approaches with liver tissue and PBMC gene expression data to distinguish among several alcohol-associated and non-alcohol-associated liver diseases simultaneously with overall classification accuracies above 75%. Optimization of gene feature selection played a key role in attaining high accuracies. We have also identified gene signatures that were enriched for various inflammation and metabolism pathways, which thus show promise as diagnostic biomarkers for the liver diseases included in the study.

We found that the use of feature selection was one of the most crucial components of successful classification. The feature space of a typical RNA-seq experiment consists of thousands of genes. While exploring every possible subset of genes is computationally infeasible, we found that it was crucial to experiment with at least a small number of intelligently selected gene subsets. The filter feature selection proved to be the most effective and runtime efficient approach. While DE and IG filters attained similar classification accuracies, the DE filter resulted in more biologically relevant gene sets. The choice of ML classifier had minor impact on classification accuracy with LR, kNN, and SVM classifiers proving to be most effective for our datasets.

The outlier feature removal proved useful toward establishing adequate *in silico* biological relevance. Small sample size RNA-seq datasets are typically noisy and highly impacted by batch effects. RNA-seq data also often contains many aberrantly expressed non-coding genes. The removal of these genes resulted in gene signatures with more biologically relevant terms. In addition to using Enrichr for *in silico* biological validation, we also performed pathway analysis of best gene signatures for the 5-Way datasets using IPA, GSEA, and BTM analysis software, which highlighted relevant pathways in these gene sets on pairwise comparison basis (Tables S28–S32).

Using the best gene signature identified in the PBMC 5-Way dataset (AH, Healthy, AC, NAFLD, HCV), we examined significantly enriched pathways with IPA for each pairwise comparison. The significantly enriched pathways mainly fell into 2 categories: iron homeostasis and immune system processes. Iron homeostasis pathways included heme biosynthesis, tetrapyrrole biosynthesis, and erythropoietin signaling. Iron homeostasis is one of the principal liver functions, while most of the functional iron in the body is stored in hemoglobin within red blood cells. Large amounts of iron are recycled from senescent erythrocytes by macrophages.^27^ Chronic liver disease has been extensively linked to iron deficiency anemia.^28^ Therefore, it would be expected that PBMCs demonstrate altered expression of genes that play crucial roles in iron homeostasis in patients with chronic liver diseases. Erythropoietin plays a crucial role in regulation of erythropoiesis and has been shown to ameliorate fatty liver disease in animal models.^29^ Immune system processes included signaling pathways (*e.g.*, TREM1, IL-8, IL-17A, B cell receptor, and acute phase), complement system, and agranulocyte adhesion and diapedesis. TREM1 expression in resident and infiltrating immune system cells promotes inflammation during the course of liver disease.^30^ The IL-8 signaling pathway is enriched by differential expression of the *CXCR1* gene within the PBMC 5-Way dataset. Altered expression of CXCR1 in circulating monocytes of patients with cirrhosis has previously been established.^31^ Increased expression of IL-17A within a range of immune cells has previously been shown to be an indicator of chronic liver disease.^32^ In addition to pathway analysis with IPA, we also performed GSEA and BTM analyses of the PBMC 5-Way best gene signature. The most enriched GSEA pathways per pairwise comparison reflected immune response and homeostatic processes (Table S31). Differentially enriched BTMs primarily involved immune response, inflammatory response, oxygen transport, and hemopoiesis (Table S32). Thus, the results of the GSEA and BTM analyses provided additional confirmation of the IPA analysis, and insights into the directionality of the enriched pathways. While alterations in the expression of immune and inflammatory genes in PBMCs due to liver diseases were expected, it was intriguing that the expression levels of these genes could be used to differentiate between these diverse liver diseases.

Pathway analysis of the Liver 5-Way dataset identified many pathways related to metabolism, biosynthesis, and degradation. For example, when comparing disease groups in the liver dataset (AH, AC, NAFLD, HCV) to healthy controls, some commonly and significantly enriched pathways involved degradation of bupropion, methylglyoxal, tryptophan, acetone, nicotine, and melatonin. Retinoate, retinol, and estrogen biosynthesis pathways were also highly enriched. Abnormal estrogen metabolism due to liver disease has been established previously.^33^ Abnormal vitamin A metabolism has been heavily implicated in liver disease, especially NAFLD.^34^^,^^35^ The retinoate and retinol pathways were enriched by differential expression of aldo-keto reductase family 1 member B10 (AKR1B10). AKR1B10 has been reported as an effective biomarker of advanced liver fibrosis and liver cancer.^36^^,^^37^ The pregnane X receptor activation pathway was also highly enriched across many pairwise comparisons and has been implicated in chronic liver disease.^38^ The pairwise comparisons involving AH and AC conditions were enriched for ethanol degradation pathways^39^ by differential expression of CYP2A7 in our gene signature. Changes in expression of CYP2A genes in liver tissue have been linked with NAFLD and alcohol-associated liver disease.^40^ These enriched pathways and genes suggest that alterations in the liver’s ability to degrade and synthesize these compounds may be related to the liver diseases in the study.

Both PBMC 5-Way and LV 5-Way datasets were enriched for several common immune system pathways, such as: inhibition of matrix metalloproteases (MMPs), macrophage migration inhibitory factor regulation of innate immunity, and interferon signaling pathways. As reported by IPA, these pathways were enriched by *MMP8, PLA2G4C,* and *IFITM3* genes, respectively, in the PBMC 5-Way dataset. In the LV 5-Way dataset, these pathways were enriched by *MMP7, PLAG2GA*, and a combination of *IFITM1, IFI6,* and *ISG15* genes, respectively. Genes in the MMP family have been established as key actors in liver regeneration and fibrosis.^41^ PLA2G4C has been reported to play a role in HCV replication.^42^ Interferon genes have long been implicated in both HCV and viral infections broadly.^43^ As expected, the interferon signaling pathway had higher enrichment in pairwise comparisons involving HCV in both the PBMC and liver tissue datasets.

We further analyzed the gene expression data from the 19 participants with AH who donated both liver tissue and PBMCs. We identified several genes and gene families that were similarly up- or downregulated within both AH sample types, when compared with healthy controls (Table S34, Fig. S12-S14). The genes fell into 4 groups: interferon (*IFITM1, IFI44L*), MMP (*MMP7, MMP8, MMP14*), iron homeostasis (*SLC25A37, SLC11A1*), and tumor necrosis factor (*TNFS10, TNFRSF21, TNFSF13B*) genes. Notably, these findings are similar to our results when comparing the best gene sets across 5-Way PBMCs and 5-Way LV datasets. The similarities in directionality of gene expression between liver and PBMC samples lend credence to using blood-based biomarkers for AH.

While we achieved excellent classification performance and the identification of biologically relevant gene signatures, there were several limitations to our study. Use of independent datasets is crucial in ML and biomarker discovery, however, we could not find any publicly available data on gene expression in PBMCs attained from individuals with AH or AC. Therefore, only our liver tissue dataset results could be independently validated at this time. A larger study with more samples is necessary to validate the biomarkers identified. Our classification performance could also be improved with the use of more advanced feature selection methodologies such as multi-objective genetic algorithms.^44^

In conclusion, our machine learning approach using gene expression data from PBMCs and liver tissue was effective at distinguishing among multiple liver diseases and healthy controls. Additionally, our models were able to distinguish between clinically similar alcohol-associated liver conditions, such as AH and AC. Notably, the AC group for our PBMC samples included both recently drinking and abstinent individuals with AC. AC in patients reporting recent drinking is especially difficult to distinguish from AH clinically, which further demonstrates the utility of this study. While the gene expression data from liver tissue had better classification performance than that of PBMCs, the attainment of liver biopsy is difficult and not standard of care at many healthcare facilities. PBMCs from blood samples, on the other hand, can be easily attained and stored. Based on the outcome of this study, we have demonstrated that blood-based biomarkers from gene expression can be utilized with machine learning methods for the diagnosis of liver disease, paving the way toward the clinical application of liquid biopsy.

## Financial support

Funding for this study was provided to the researchers in the Southern California Alcoholic Hepatitis Consortium (SCAHC) by the 10.13039/100000027National Institute on Alcohol Abuse and Alcoholism (NIAAA) award numbers: U01AA021838 (Norden-Krichmar), U01AA021886 (Morgan), U01AA021884 (Morgan), and U01AA021857 (Liu).

## Authors’ contributions

Study concept and design (SL, TRM, TMNK); enrolled participants and collection of samples (AA, TRM, AS, JT); data curation and formal analysis (SL, CM, ZXL, TMNK); analysis and interpretation of data (SL, CM, AA, AS, JT, ZXL, TRM, TMNK); resources (AA, AS, JT, ZXL, TRM, TMNK); software design and implementation for bioinformatics, statistical, and machine learning analyses of the RNA-seq data (SL); software for initial preprocessing, data cleaning, and alignment pipeline (CM); validation (SL, TMNK); supervision (TRM, TMNK); visualization (SL); writing original draft (SL); writing – review and editing (all authors).

## Data availability statement

The human RNA raw sequencing data in this study requires deposit into dbGaP with controlled access. The public RNA data used for validation in this study is available in the GEO database under accession number GSE142530 (https://www.ncbi.nlm.nih.gov/geo/query/acc.cgi?acc=GSE142530).

## Conflict of interest

T.R.M. has received grant/research support from 10.13039/100006483AbbVie, 10.13039/100005564Gilead, Genfit, and 10.13039/100004334Merck. The remaining authors have nothing to disclose.

Please refer to the accompanying ICMJE disclosure forms for further details.
