# Supplementary material for: Differentiating between liver diseases by applying multiclass machine learning approaches to transcriptomics of liver tissue or blood-based samples
Source: JHEP Rep. 2022 Aug 18;4(10):100560. doi: 10.1016/j.jhepr.2022.100560 (PMC9472076; doi:10.1016/j.jhepr.2022.100560)
Supplement: Multimedia component 1 [file mmc1.pdf]

Differentiating between liver diseases by applying multiclass machine learning approaches to transcriptomics of liver tissue or blood-based samples

Stanislav Listopad, Christophe Magnan, Aliya Asghar, Andrew Stolz, John A. Tayek, Zhang-Xu Liu, Timothy R. Morgan, Trina M. Norden-Krichmar

Table of contents

1. Supplementary methods 5
a. Inclusion and Exclusion Criteria: 5
Alcohol-associated Liver Disease (AH, AC) Donors: 5
Specific to Alcohol-Associated Hepatitis Donors (AH): 5
Specific to Alcohol-Associated Liver Cirrhosis Donor (AC): 5
Non-Alcohol-Associated Fatty Liver Disease Donors: 6
Chronic Hepatitis C Donors: 6
Healthy Donors: 7
b. Reference Genome: 7
c. Gene Annotations: 7
d. Short-read alignment to reference genome and transcriptome: 8
e. Sample Sequencing: 8
f. Read Trimming & Quality Filters: 8
g. Sample Decontamination: 10
h. Normalized RNA-seq counts before and after application of log transformation: 11
i. Alignment Pipeline Selection: 12
j. Nested Cross-Validation Setup: 13
k. Feature Selection Strategies: 14
l. Differential Expression (DE) Feature Selection: 16
m. Information Gain (IG) Feature selection: 16
n. Feature Sizes: 16
o. Performance Metrics: 17

p. Machine Learning Classifiers. 17
q. Sample Size Calculation: 18
r. Enrichr Libraries: 18
s. Regular Expression (Regex) Patterns for Enrichr Libraries: 19
t. Impact of Outlier Gene (Feature) Removal – Variance, Intersection, and Union Filtering: 20
u. Summary of Methods: 24
v. Candidate Gene Sets: 25
w. Best Gene Set Selection: 25
x. Additional in silico biological validation methods. 26
Ingenuity Pathway Analysis (IPA): 27
Gene Set Enrichment Analysis (GSEAPreranked): 27
Blood Transcription Module (BTM) Analysis (BloodGen3Module): 28
y. Codebase: 28
2. Supplementary results 28
a. Best Gene Sets Fold Changes. 28
i. LV 5-Way. 28
ii. PBMC 5-Way. 29
b. Classification Performance, In Silico Biological Validation, and Top Enrichr Hits Tables. 31
i. LV 2-Way. 33
ii. LV 3-Way. 38
iii. LV 5-Way. 40
iv. PBMC 5-Way. 42
c. Per Replicate RNA-seq Count Heatmaps: 44
i. LV 2-Way. 45
ii. LV 3-Way. 45
iii. LV 5-Way. 46
iv. PBMC 5-Way. 46
d. Comparison of additional in silico biological validation approaches: 47
i. IPA. 47
ii. GSEAPreranked. 52
iii. Blood Transcription Module analysis (BloodGen3Module). 55
e. Misclassified Sample Analysis: 57
f. AH PBMC-LV Analysis: 58
Supplementary references 62

List of abbreviations:

AC: alcohol-associated cirrhosis (2-letter sample code)

ADA: Adaptive Boosting Algorithm

AH: alcohol-associated hepatitis (2-letter sample code)

BMI: body mass index

BTM: blood transcription module

CT: healthy controls (2-letter sample code)

DE: differential expression

DF: Maddrey's discriminant function

DT: Decision Tree Classifier

FPKM: fragments per kilobase of exon model per million reads mapped

FS: feature selection

GNB: gaussian naïve bayes

GSEA: Gene Set Enrichment Analysis

HCV: hepatitis C virus

HP: chronic hepatitis C viral infection (2-letter sample code)

IG: information gain

IPA: Ingenuity Pathway Analysis

IRB: institutional review board

kNN: k-nearest neighbors

LR: logistic regression

LTCDS: Liver Tissue Cell Distribution System

LV: liver (tissue name)

MCC: Matthew's Correlation Coefficient

MELD: Model for End-Stage Liver Disease

ML: machine learning

NAFLD: non-alcohol-associated fatty liver disease

NF: non-alcohol-associated fatty liver disease (2-letter sample code)

PBMC: peripheral blood mononuclear cells

RF: random forest

RNA: ribonucleic acid

RNA-seq: RNA sequencing

SCAHC: Southern California Alcoholic Hepatitis Consortium

SFS: sequential feature selection

SVM: support vector machine

1. Supplementary methods

Sections **a-j** below describe the collection and processing of the samples that were RNA sequenced in the current study.

The study was approved by the Department of Veterans Affairs VA Long Beach Healthcare Systems Institutional Review Board (IRB# 1254), by the Human Subjects Committee, Los Angeles Biomedical Research Institute (Project No. 20607-0), University of Southern California Health Sciences Campus Institutional Review Board (Project # HS-13-00815), and by the University of California, Irvine Institutional Review Board, HS #2016-3064. All participants signed written consents prior to providing biospecimens.

For information about the independent RNA-seq liver tissue dataset used for external validation, please refer to GSE142530 (1).

a. Inclusion and Exclusion Criteria:

Alcohol-associated Liver Disease (AH, AC) Donors:

Common Inclusion Criteria: History of chronic alcohol consumption sufficient to cause liver damage. Generally, this is considered to be >40 g/day for women and >60 g/day for men, for many years.

Common Exclusion Criteria: Liver disease significantly caused by hemochromatosis, autoimmune liver disease, Wilson disease, NAFLD, hepatitis C, or hepatitis B.

Specific to Alcohol-Associated Hepatitis Donors (AH):

Inclusion Criteria: A clinical diagnosis of possible alcoholic hepatitis. Serum total bilirubin >3 mg/dL.

Specific to Alcohol-Associated Liver Cirrhosis Donor (AC):

Inclusion Criteria: This group contained both abstinent and recently drinking alcohol associated cirrhosis. Inclusion Criteria for Abstinent donors: Abstinent (consumption of less than one standard drink*/week) during the 6 months prior to enrollment. Inclusion Criteria for Recently drinking donors: Heavy alcohol use until recently (stopped/reduced alcohol use within past 60 days). For the current study, both groups were combined into a single group for analysis.

Non-Alcohol-Associated Fatty Liver Disease Donors:

Inclusion Criteria: A clinical diagnosis of non-alcoholic fatty liver disease (NAFLD) with at least two of the following criteria: a) A history of diabetes mellitus or use of medicines to treat diabetes (e.g., metformin, insulin, etc.) b) Liver biopsy consistent with NAFLD or NASH c) BMI>30 d) Fasting triglycerides >250 mg/dL or receiving treatment for high triglycerides e) CT or MRI imaging consistent with NAFLD ALT >50 IU/ml at baseline. Abstinent (consumption of less than one standard drink*/week) during the 6 months prior to enrollment.

Exclusion Criteria: Liver disease caused by hemochromatosis, autoimmune liver disease, Wilson disease, hepatitis C, or hepatitis B. Participants currently receiving treatment for NAFLD.

Chronic Hepatitis C Donors:

Inclusion Criteria: Chronic hepatitis C diagnosis. Evidence of cirrhosis based on at least one of the following criteria: a) Fibroscan stiffness >12.5 kPa b) Liver biopsy showing Metavir F3 or F4 or Ishak fibrosis stage 4, 5, or 6 c) Nodular liver on ultrasound, CT or MRI d) FIB-4 score >3.25 e) Platelet count <150,000 /mm^3. Abstinent (consumption of less than one standard drink*/week) during the 6 months prior to enrollment.

Exclusion Criteria: Clinical evidence for NAFLD or laboratory evidence of hemochromatosis, autoimmune liver disease, Wilson disease, or hepatitis B. Has received or currently receiving treatment for HCV infection.

Healthy Donors:

Inclusion Criteria: AUDIT-C scores of <4 for men and <3 for women (signifying no alcohol misuse). Abstinent (consumption of less than one standard drink*/week) during the 6 months prior to enrollment.

Exclusion Criteria: Clinical history or laboratory evidence of liver disease including alcoholic liver disease, NAFLD, hemochromatosis, alcoholic hepatitis, autoimmune liver disease, Wilson disease, hepatitis C, or hepatitis B. BMI>32. Any of the following laboratory abnormalities within 90 days prior to signing the consent. - Creatinine: >1.5 mg/dL; - Hemoglobin: <12 g/dL; Total bilirubin: >1.5 mg/dL; - AST: >40 IU/mL; - ALT: >40 IU/mL.

b. Reference Genome:

To determine if the reference genome influenced our results, gene expression analyses were performed using both the hg19 (GRCh37 assembly) and hg38 (GRCh38 assembly) human reference genomes, downloaded from the UCSC Genome Browser. In both cases, chrM was not included in the assembly.

c. Gene Annotations:

For each reference genome, we performed the gene expression analyses using four distinct sets of gene annotations for comparison purposes. In particular, we used the following four versions of the gene annotations: 1) RefSeq from the UCSC Genome Browser (Dec 2017); 2) GENCODE release 28 (Apr 2018); 3) Ensembl release 91 (Dec 2017); and 4) a merged set of gene annotations curated from these versions of RefSeq, GENCODE, and Ensembl annotations.

d. Short-read alignment to reference genome and transcriptome:

The filtered and decontaminated reads were aligned to the reference genome and transcriptome for each of the 8 combinations of reference genome and gene annotations described in the previous sections. Three short-read aligners were used during this step for comparison purposes: 1) TopHat release 2.1.1. (2) in combination with Bowtie2 2.3.4.1 (3) with default settings (TUXEDO); 2) HiSat2 2.1.0 (4) with default settings (HISAT2); and 3) STAR 2.6.0 (5) with default settings (STARCQ).

e. Sample Sequencing:

RNA was isolated from the cell pellets and liver tissue according to total RNA extraction kit instructions (Qiagen RNAeasy kit). Total RNA was monitored for quality control using the Agilent Bioanalyzer Nano RNA chip and Nanodrop absorbance ratios for 260/280nm and 260/230nm. Library construction was performed according to the Illumina TruSeq mRNA stranded protocol.

All samples included in this study were RNA sequenced on an Illumina platform by the Genomics High-Throughput Facility (GHTF) at the University of California, Irvine (UCI), except for one healthy liver sample for which the sequencing data was directly downloaded from the European Bioinformatics Institute (EBI) ArrayExpress database (accession number E-MTAB-1733) (6). The number of paired or single reads per sample was approximately 140M before filtering and decontamination.

f. Read Trimming & Quality Filters:

The sequencing reads in each dataset were first filtered to remove low quality reads and trim all 3' regions matching with the Illumina sequencing primers or 5' regions with skewed base distributions. The following is each step of the protocol:

1) Sequencing primers attached to short inserts were removed using Trimmomatic release 0.38 (7).

2) Reads not passing the standard Illumina quality tests as reported in the header line of each entry in a FastQ file were removed.

3) Reads with any number of uncalled bases (N) were discarded with a few exceptions for some positions observed with more than 3% uncalled bases in the corresponding dataset. In these cases, 1 uncalled base max was allowed in the reads.

4) Reads were trimmed on the 5' end to remove the positions observed with highly variable base distribution following this protocol. First, the standard deviation of the base distribution was calculated for each position. Second, the mean standard deviation was calculated for every contiguous set of 5 positions in the reads. Finally, positions on the 5' end were trimmed as long as the mean standard deviation of the first 5 bases in the reads was greater than twice the lowest mean standard deviation observed in the reads during the previous step. In most cases, 5 to 15 positions were trimmed on the 5' end of the reads in each dataset following this protocol.

5) Reads were trimmed on the 3' end using a fixed number of positions = 1 except for three datasets for which between 25 and 30 positions were trimmed on the 3' end to account for sequencing issues specific to these samples.

6) Reads shorter than 60 bases after trimming were discarded from the datasets.

7) A min PHRED quality score per position of 20 was used to further filter the reads with several positions allowed below this threshold ranging from 2 to 10 such that the lowest number of exceptions not discarding more than 20% of the reads was selected. No more than 10 exceptions were allowed during this step.

8) A min average PHRED quality score per read was used as an additional filter, with a value ranging from 24 to 36 such that the highest mean quality score not discarding more than 20% of the reads was selected.

On average, 9.62% of the original reads were discarded during this step and 15.43% of the paired reads were orphaned. The mean PHRED quality score of the remaining reads was approximately 40.

g. Sample Decontamination:

The remaining quality-filtered and trimmed reads for each dataset were then further filtered to remove possible contaminants in each sample such as PhiX control reads or bacterial contamination. In addition, both the human mitochondrial genome and ribosomal DNA/RNA sequences were treated as contaminants during this step due to highly variable quantities of these reads in the various datasets generated during the experiment, ranging from a few percent of the reads in most cases to about 80% of the reads for some highly contaminated samples. Such differences significantly impact gene expression results, notably the FPKM values calculated during the next step of our analysis, so this bias was removed prior to the gene expression analysis by simply removing the corresponding reads from all the datasets. This step was performed using the following protocol. The reads were first aligned to all the contaminant sequences using Bowtie2 release 2.3.4.1. Any read successfully located on any contaminant sequence was then aligned against the human transcriptome using the same short-read aligner. Reads not matched with any known human transcript (i.e. only matched to a contaminant sequence) and reads with a better alignment score to a contaminant sequence than the best alignment score with the human transcriptome were discarded, the remaining reads were kept for

the rest of the analysis. On average, approximately 115M paired and single reads were left per sample and used for the gene expression analysis described in the next sections.

h. Normalized RNA-seq counts before and after application of log transformation:

Fig. S1 shows the relationship between variance and mean of the RNA-seq counts for the PBMC Alcoholic Hepatitis (AH) samples. It can be readily observed that there is a linear relationship between the two. This is usually an undesirable property for machine learning (ML) algorithms. After transforming the RNA-seq counts using the ln(1+count) formula we can observe that there is no longer a linear relationship between mean and variance of the RNA-seq counts (Fig. S2). Moreover, the variance and mean values are much smaller and more consistent. The log transformation improved the classification accuracy by approximately 5% for logistic regression classifier when tested with LV 2-Way dataset. Therefore, we used log transformed counts with all four of our datasets.

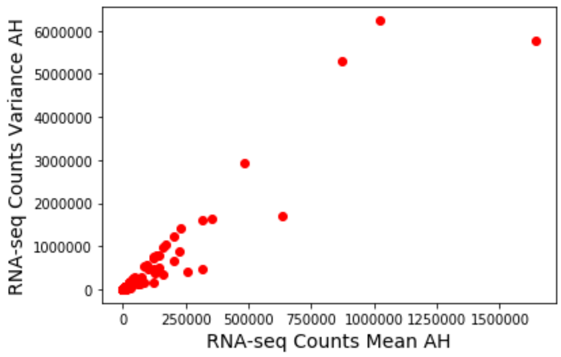

Fig. S1: Geometrically Normalized RNA-seq counts.

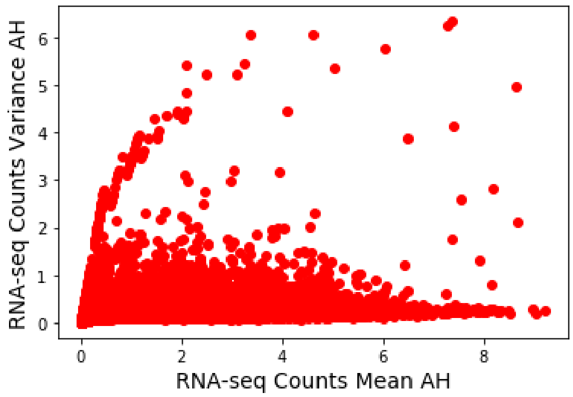

Fig. S2: RNA-seq counts above after being transformed using ln(1+count) formula.

i. Alignment Pipeline Selection:

We compared the results from 24 different alignment pipelines using the PBMC AH and CT conditions. These 24 pipelines were formed using two human reference genomes (hg19, hg38), four different genome annotations (Curated, Ensembl, Gencode, and Refflat), and three different genome aligners (Tuxedo, Hisat2, and Starcq). The PBMC AH and CT counts from each of the genome pipelines were then utilized in our classification and feature selection pipeline using differential expression feature selection only. No alignment pipeline proved to be advantageous over others according to classification performance. We then compared the alignment pipelines according to the in silico biological validation of the selected genes utilizing Ingenuity Pathway Analysis (IPA) software (Fig. S3). Ensembl annotation resulted in the most biologically relevant

genes according to IPA. The choice of human reference genome and aligner did not seem significant and therefore we decided to utilize the more recent hg38 reference genome and Starcq aligner along with Ensembl annotation for our four datasets.

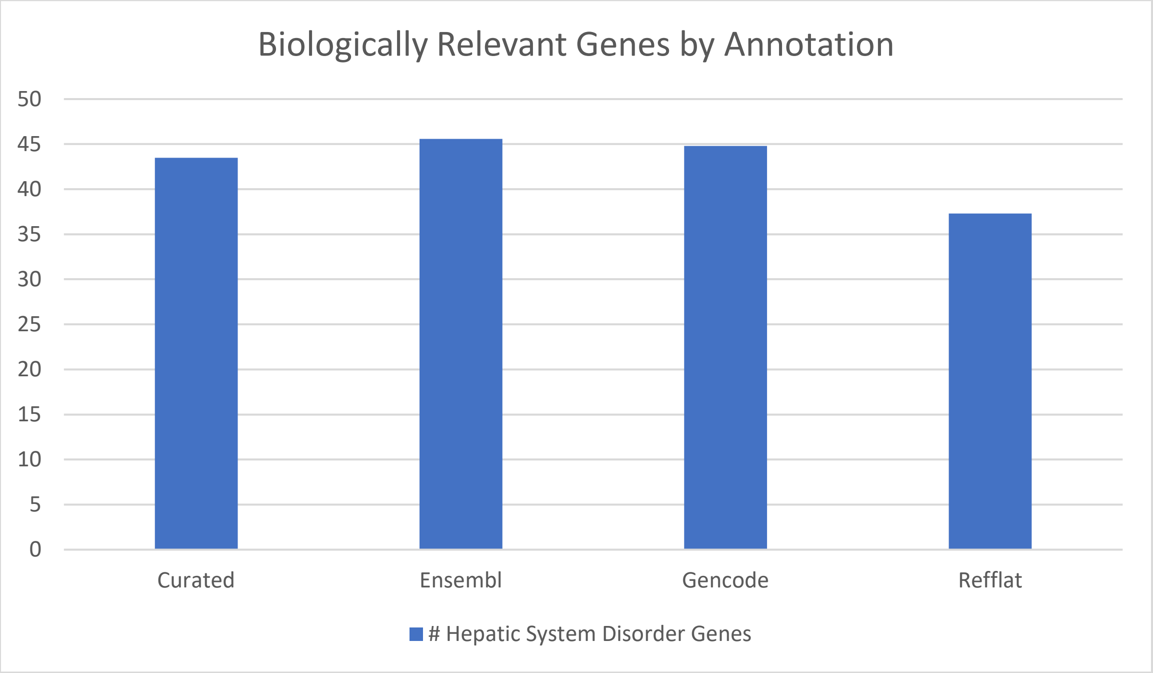

Fig. S3: Comparison of annotations by number of hepatic system disorder related genes using the PBMC 2-Way dataset.

j. Nested Cross-Validation Setup:

We utilized nested cross-validation to attain the estimates of classification performance for various feature selection (FS) strategies, classifiers, and feature sizes within our data. The best feature (gene) sets selected for each of the four datasets were then validated in the independent test set. The nested cross-validation was implemented in the standard configuration with k = 5 in both the inner and outer loops. The outer loop was used for model evaluation (i.e., classification performance), while the inner loop was used for model selection (i.e., hyper-parameter tuning). The feature selection was done within both inner and outer loops. That is FS was done for each training set in inner and outer loops. This means that effectively there were 30 training sets (25 in

inner loop, 5 in outer loop) as part of a single nested cross-validation execution. Feature selection occurred for each of these training sets.

Since one of our classification strategies relied on differential expression as computed by Cuffdiff (8), the feature selection process within nested cross validation was time consuming. A single Cuffdiff analysis could require anywhere from 30 minutes to 5 hours depending on the number of samples. In order to keep runtime reasonable, all folds were pre-defined, and only a single splitting of samples into folds (for both inner and outer loops) was used within each dataset. Typically, multiple repeated data splits of samples to folds are desired to obtain best estimate of classifier's performance. However, due to Cuffdiff's large runtime performing multiple data splits proved to be prohibitive.

Cuffdiff produced three key files: genes.read_group_tracking containing the normalized RNA-seq counts, gene_exp.diff containing the differential expression analysis data over all input samples, and the read_groups.info containing the names of input CXB files (samples). CXB files (samples).

k. Feature Selection Strategies:

Feature selection for gene expression data was essential, since our datasets contained tens of thousands of genes, far more than the number of samples. ML algorithms typically perform very poorly when given significantly more features than samples. Initially, we briefly compared three types of feature selection strategies within our study: filter feature selection via differential expression (DE) and information gain (IG) algorithms, hybrid feature selection (filter + wrapper), and embedded feature selection via random forest (RF) algorithm. All three strategies resulted in similar classification performance (Table S1). However, the filter feature selection had much lower runtime then the hybrid and embedded FS strategies. Additionally, we had

concerns that both hybrid and embedded feature selection strategies were prone to overfitting based on past analyses. Therefore, we decided to use only the filter feature selection strategies within the remainder of the study.

Table S1: Comparison of three feature selection architectures: filter, hybrid, and embedded using LV 2-Way dataset.

Feature Size	Filter: DE	Hybrid: DE + SFS	Embedded: RF
2	0.91	0.88	0.95
3	0.95	0.97	0.91
4	0.97	0.88	0.95
5	0.97	0.95	0.95
10	0.97	0.97	0.95
15	0.97	1.0	0.97
20	1.0	0.97	1.0

*Used LR classifier with Filter and Hybrid architectures. Used Union filter with threshold of 3.0 with all architectures.

The hybrid feature selection was done by pairing the filter feature selection strategies (DE, IG) with forward sequential feature selection algorithm (forward-SFS) as described in scikit-learn documentation. The features were first selected by filter feature selection and then halved using forward-SFS. The forward-SFS was performed using logistic regression classifier.

The embedded feature selection was performed using random forest. Specifically, the RF classifier was simply given data with all features included. We then extracted the feature rankings from the RF models to determine which features it valued the most.

l. Differential Expression (DE) Feature Selection:

For every training set all pairwise comparisons (within gene_exp.diff files) were filtered by normalized FPKM (> 1.0) and q-values (< 0.05). All of the genes belonging to each pairwise comparison were then sorted by absolute log2(fold change) value, and the top gene for each pairwise comparison was taken. If that gene was not already in the top genes list, the gene was added to the list. The algorithm continued to cycle through the pairwise comparisons until the desired number of genes was reached. This procedure was used for all the datasets. The best features for each training set were then stored in text files.

Other DE feature selection approaches were implemented and tested by us as well. However, we found that pairwise DE selection was best performer since other DE feature selection approaches, we tested were too easily biased by the most strongly differentially expressed pairwise comparisons.

m. Information Gain (IG) Feature selection:

For every training set, the genes within normalized RNA-seq counts were ranked using the scikit-learn's mutual_info_classif function.

n. Feature Sizes:

We refer to the number of features selected during filter feature selection as "feature size". The feature sizes used with DE & IG feature selection were: 2, 3, 4, 5, 10, 15, 20, 25, and, 50 for LV 2-Way dataset and 10, 25, 50, 100, 150, 200, 250, 300, 350, 400, 450, and 500 for the other three datasets. The feature sizes denote the number of features selected within each training set. We found during preliminary testing that we required at least 5-10 features per training set to attain reasonable classification performance and that we generally did not see benefit in using more than 500 features per training set. The maximum feature size was also influenced by our power

size calculation (that is number of significantly differentially expressed genes within our datasets).

o. Performance Metrics:

Several different ML performance metrics were evaluated for use in this project including overall accuracy, per-class accuracy, balanced accuracy, confusion matrices, Matthews Correlation Coefficient (MCC), and F1-score. Balanced accuracy, MCC, and F1-score attempt to account for class sizes when evaluating performance, while the confusion matrices provide information about both class sizes and also per-class accuracies. Therefore, we chiefly reported our classification performance in the form of confusion matrices.

p. Machine Learning Classifiers.

We initially tested 7 classifiers: Adaptive Boosting Algorithm (ADA) using decision tree, decision tree (DT), gaussian naïve bayes (GNB), logistic regression (LR), k nearest neighbors (kNN), support vector machine (SVM), and random forest (RF). Based upon comparison of their performance and run time, we narrowed down our selection to LR, kNN, and SVM only. Table S2 demonstrates the performance of all classifiers using the LV 2-Way dataset with filter feature selection, with the exception of RF, which belongs to embedded feature selection architecture.

Table S2: Comparison of six ML classifiers in LV 2-Way dataset.

	ADA	DT	GNB	kNN	LR	SVM
2	0.77	0.79	0.84	0.82	0.82	0.82
3	0.92	0.79	0.92	0.86	0.9	0.86
4	0.9	0.93	0.94	0.97	0.88	0.95
5	0.97	0.85	0.95	0.95	0.97	0.92
10	0.95	0.95	0.97	0.97	0.97	0.95
15	0.97	0.93	0.97	0.97	0.97	0.95
20	0.93	0.93	0.97	0.97	0.97	0.95

*Classifiers in Table S2 were used in conjunction with DE feature selection and Intersection filter with threshold of 3.0.

q. Sample Size Calculation:

There are few established guidelines for calculating sample size for RNA-seq experiments. Recommendations vary from having at least 3, 6, or 12 biological replicates per condition depending on sequencing depth and fold change cutoff. All selected conditions within our PBMC dataset contain more than 12 biological replicates. All selected conditions within the liver dataset contain more than 6 biological replicates. The average number of reads per sample is approximately 115 million after filtering and decontamination. We utilized the RNASeqPower R package (9) to establish the best fold change cutoff and the expected number of significantly differentially expressed genes (SDEGs) for our LV 2-Way dataset. According to the output of the package for our dataset, there are approximately 450 SDEGs in the LV 2-Way dataset. We assumed that the number of SDEGs is approximately similar across all datasets. This helped us to determine the upper bound on useful feature sizes.

r. Enrichr Libraries:

The genes selected during feature selection were computationally evaluated using gene enrichment analysis via Enrichr (10) with pathway, tissue, and disease Enrichr libraries listed below. Custom code was written using regular expressions to match: a) immune system pathways; b) cell types that comprise blood and liver tissues; c) diseases included the conditions within this study (AH, AC, NAFLD, HCV) along with several other liver and blood disorders.

In order to attain the top three Enrichr hit tables (Tables S18, S21, S24, and S27) we performed the following steps. Enrichr hits for the best gene sets, after matching using the regular expressions, were sorted by adjusted p-value with a cutoff of 0.05. We removed entries with redundant term names or genes. We then displayed up to three top entries for each category: pathway, tissue, disease.

Enrichr Libraries used:

Pathways: 'BioPlanet_2019', 'WikiPathways_2019_Human', 'KEGG_2019_Human', 'GO_Biological_Process_2018'.

Tissues: 'ARCHS4_Tissues', 'Human_Gene_Atlas'.

Diseases: 'Disease_Perturbations_from_GEO_up', 'Disease_Perturbations_from_GEO_down'.

s. Regular Expression (Regex) Patterns for Enrichr Libraries:

The regular expression (regex) patterns used for filtering the results returned by Enrichr are listed below.

Disease Regex:

'hepa|liver|cirrhosis|NAFLD|liver fibrosis|NASH|steatohepatitis|HCV|alcohol|sepsis|septic shock|hypercholesterolemia|hyperlipidemia|obesity'

Tissue Regex:

'Blood|Macrophage|Erythro|Platelet|Basophil|Neutrophil|Eosinophil|Cytokine|Tumor Necrosis Factor|Monocyte|Lymphocyte|Granulocyte|Dendritic|Megakaryocyte|T Cell|B Cell|NK Cell|Toll-like receptor|Fc receptor|Liver|Hepatocyte|Stellate|Kupffer|Sinusoidal Endothelial Cells|CD34+|Natural Killer Cell|PBMC|Tcell|Bcell|lymphoblast|CD8+|CD19+|CD4+|CD71+|Omentum'

Pathway Regex:

'Interferon|Immun|Interleukin|Prolactin|Complement|Chemokine|Oncostatin M|Rejection|Inflamma|IL1|IL-

|selenium|osteopontin|circulation|coagulation|clotting|biosynthesis|degradation|cholesterol|lipid|TNF|steroid|metal ion|heme|metallo|CXCR|LDL|Phagocytosis|metabolism|TYROBP|AP-1|'

Additionally, the pathway regex included all of the disease and tissue terms.

t. Impact of Outlier Gene (Feature) Removal – Variance, Intersection, and Union Filtering:

RNA-seq serves as a proxy for the level of gene expression in a biological sample. One challenge with interpretation of RNA-seq output, however, involves expression of non-coding genes that were presumed to be removed via poly(A)-selection. It is also common to observe genes with aberrant expression that poorly distinguish between the study conditions, thereby hindering classification performance. As an example, in Fig. S4 the RNA-seq counts of the LV 2-Way dataset are visualized as a heatmap. The genes selected were chosen by differential expression analysis. We observed that genes such as SNHG25, RNY1, RNU6ATAC, and UBA3 are all highly variant. Moreover, three of these are non-coding. The Fig. S5 shows the same dataset after genes were filtered using the Union filter with threshold of 3.0. In this example, the genes removed were replaced with other top DE genes such that the total number of genes remained the same. The latter heatmap is much more visually distinct between the AH and CT conditions.

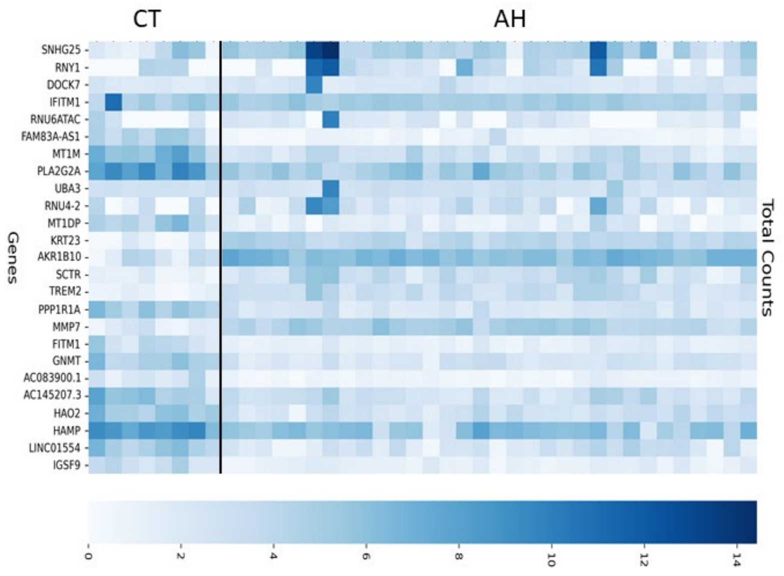

Fig. S4: LV 2-Way RNA-seq counts – no filter, 25 genes total.

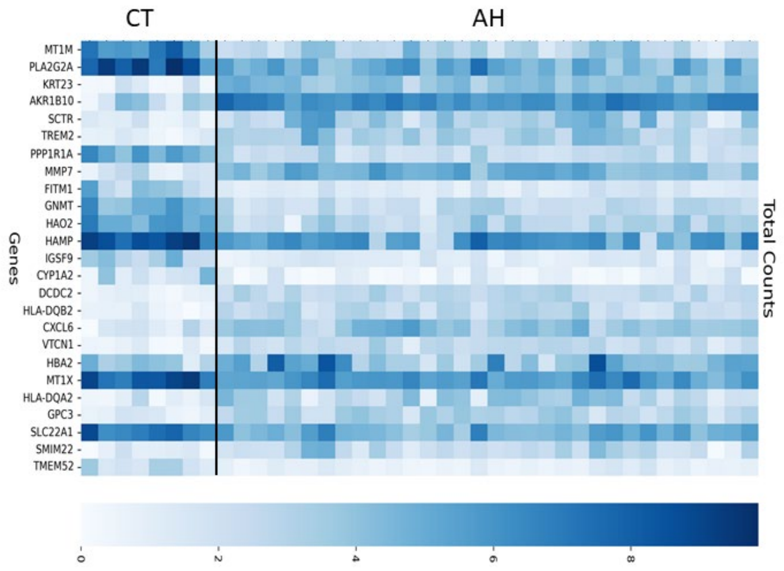

Fig. S5: LV 2-Way RNA-seq counts – Union filter with threshold of 3.0, 25 genes total.

Based on our observations and explanation above, we developed three strategies for removing undesirable genes: Variance, Intersection, and Union filtering. Variance filtering was implemented by removing genes in which the RNA-seq counts for at least one sample were

further than a standard deviation multiplied by the threshold from the mean in any of the conditions (AH, CT, etc.). Throughout the study, we used three threshold values: 2.5, 3.0, and 3.5. Lower thresholds resulted in more genes being eliminated, while higher thresholds resulted in less genes being eliminated. The filtered-out genes were not used in the subsequent feature selection process. The Union filter built upon the Variance filter by removing all genes that were either highly variant (as defined above) ***or*** non-coding as determined by ENSEMBL database's gene "biotype" column. The Intersection filter was similar to the Union filter, except that only the genes that were both highly variant ***and*** non-coding were removed. In addition to improving the odds of successful classification, the outlier feature filtering was also found to improve in silico biological validation of identified gene signatures, since protein coding genes are more extensively annotated than non-coding ones. These three filters also removed all genes whose counts were mostly zeroes across all samples.

We applied the three filter procedures (each paired with three possible threshold values of 2.5, 3.0, and 3.5, for a total of 9 filter configurations) to the LV 2-Way dataset. Tables S3 and S4 show the impact of each filtering strategy (with threshold of 3.0) on the overall classification accuracy and biological relevance of LV 2-Way dataset. The biological relevance was determined by performing gene enrichment analysis using Enrichr. The in silico biological validation results are reported as follows: pathway hits / tissue hits / disease hits. In the example below, the genes removed by the outlier filtering strategy were replaced with the next highly-ranked DE genes. The classification accuracies were attained using nested cross-validation. The feature size in the Table S3 is the feature size within each individual training set of the nested cross-validation. Before commencing with in silico biological validation, we merged the gene sets produced by training sets in the outer loop of nested cross-validation. The features sizes in

Table S4 are listed with the following notation: feature size of training set in nested cross validation – feature size of merged gene set using first filter procedure / feature size of merged gene set using second filter procedure / and so on. For example, in Table S4, the numbers in the first column are the feature size of the training set in nested cross validation, followed by the number of genes in the four filter procedures: No Filter/Variance 3.0/Union 3.0/Intersection3.0. Based on further analysis in the LV 3-Way dataset, we decided to use Intersection and Union filters with thresholds of 2.5, 3.0, and 3.5 for all four of our datasets. The gene sets of size 0 could not be analyzed using Enrichr. Therefore, cells corresponding to empty gene sets in Table S4 are labeled as NA.

Table S3: The impact of outlier feature removal strategies on classification accuracies of k nearest neighbors (kNN) classifier and DE feature selection within LV 2-Way dataset.

Feature Size	No Filter	Variance 3.0	Union 3.0	Intersection 3.0
2	0.8	0.95	0.93	0.82
3	0.76	0.97	0.95	0.86
4	0.86	0.95	0.97	0.97
5	0.89	0.95	0.97	0.95
10	0.94	0.97	0.95	0.97
15	0.97	0.97	0.95	0.97
20	0.97	0.97	0.97	0.97
25	0.97	0.97	1	0.97
50	0.97	0.97	0.95	0.97

Table S4: The impact of outlier feature removal strategies on in silico biological relevancy of LV 2-Way gene sets with DE feature selection.

Feature Sizes	No Filter	Variance 3.0	Union 3.0	Intersection 3.0
2 – 0/0/0/1	NA	NA	NA	1/0/0
3 – 1/0/0/2	1/0/0	NA	NA	16/0/12
4 – 1/0/1/2	1/0/0	NA	23/0/8	16/0/12
5 – 2/0/3/3	16/0/12	NA	30/2/4	18/0/0
10 – 5/7/9/9	34/0/3	31/2/5	28/3/6	22/1/3
15 – 11/9/9/12	13/1/4	28/3/6	28/3/6	17/2/5
20 – 13/11/13/13	1/1/5	19/3/5	1/4/5	18/3/7
25 – 16/14/14/18	1/3/7	1/4/5	1/4/6	1/3/7

50 – 31/30/34/33	1/4/7	0/4/5	1/5/12	1/3/7

*Numbers in Filter columns denote number of Enrichr hits in: pathway/tissue/disease. Higher scores for the number of pathway/tissue/disease hits suggest that the genes were more biologically relevant. NA represents the case where there were no genes in the set for input to Enrichr.

u. Summary of Methods:

While we experimented with a large number of methods with the LV 2-Way (and sometimes LV 3-Way) datasets, we were able to reduce the set of methods applied to our four datasets as shown in Table S5.

Table S5: The methods used within the study initially and as part of final configuration.

Methods	Feature Selection	Outlier Feature Removal	ML Classifiers
Briefly Examined	Filter (DE, IG), Hybrid (Filter + Wrapper), Embedded (RF).	Variance, Intersection, and Union filtering. (Standard deviation thresholds: 2.5, 3.0, 3.5)	LR, kNN, SVM, GNB, DT, ADA, RF.
Final Configuration	Filter (DE, IG).	Intersection and Union filtering. (Standard deviation thresholds: 2.5, 3.0, 3.5)	LR, kNN, SVM.

Therefore, the final analysis included the following method configurations for each of the four datasets: 2 feature selection strategies (DE, IG), 2 outlier feature removal strategies (Intersection, Union) each paired with three different thresholds (2.5, 3.0, 3.5), and 3 ML classifiers (LR, kNN, SVM). This resulted in a total of 36 configurations. For each configuration there was also a range of possible feature sizes as described in feature size section above. The nested cross-validation ML metrics were recorded for each of these configurations, for each feature size.

v. Candidate Gene Sets:

Since one of the overarching goals of this study was to identify characteristic gene expression signatures to diagnose liver disease using liver tissue and PBMC RNA-seq data, the next step of our pipeline involved selecting the best gene sets for our datasets. Within nested cross-validation, feature selection was performed for every training set in both inner and outer loops, resulting in 30 total gene sets (5 in outer, 25 in inner) for each feature size. The gene sets selected in the inner loops are not relevant, since the inner loop was only used for hyper-parameter tuning. Therefore, we developed a method of merging the gene sets produced for each of the outer loop training sets. The strategy used was as follows: if a given gene appeared in N out of the 5 (k = 5 in outer loop) gene sets it was added to the merged gene set. After examining the results, we determined that N = 4 and N = 5 yielded our best results. The candidate gene sets were analyzed using Enrichr to establish their biological relevancies. The classification accuracy attained from the associated instance of the nested cross-validation of each candidate gene set was also examined.

w. Best Gene Set Selection:

From the large collection of candidate gene sets attained by running the 36 different method configurations for each dataset across multiple feature sizes, we used the following strategy to select a single best candidate gene set for each of the four datasets. This process involved the evaluation of a combination of candidate gene set's size, classification performance, and biological relevancy metrics. The algorithm for picking best gene sets is described below.

1) The candidate gene set size was restricted between 5 (genes per pairwise comparison) to 100 total genes, if possible. Gene set sizes of between 100 and 200 were also considered, if suitable performance was not observed in candidate gene sets below 100 genes. The LV 2-Way dataset contains 1 pairwise comparison, LV 3-Way dataset contains 3

pairwise comparisons, and 5-Way datasets contain 10 pairwise comparisons. Therefore, the candidate gene set sizes, using the range guidelines above for each dataset, are as follows: 5-100 genes for LV 2-Way, 15-100 genes for LV 3-Way, and 50-100 genes for LV 5-Way and PBMC 5-Way. The gene set size guidelines were developed to minimize the chance of either under- or overfitting.

2) Biological relevancy as indicated by Enrichr was prioritized slightly higher than the classification accuracy. That is, gene sets with highest number of pathway, tissue, and disease hits were examined in detail first. Gene sets were only considered if they included at least 10 pathway hits, 1 tissue hit, and 3 disease hits. The tissue, pathway, and disease hits were examined to verify that they were appropriate and relevant to the disease groups.
3) Total and per-class classification accuracies were considered after the in silico biological relevancy. In general, only gene sets within 10% of the best recorded performance (for a given dataset) were considered.

Once a single gene set that best satisfied all 3 criteria was selected, it was used to generate the heatmaps, confusion matrices, and pathway analysis. The liver tissue gene sets selected from our data set were evaluated with the independent validation dataset.

x. Additional in silico biological validation methods.

In order to further analyze and validate our gene sets we performed additional annotation enrichment analysis using Ingenuity Pathway Analysis (IPA), Gene Set Enrichment Analysis (GSEAPreranked), and blood transcription module analysis (BloodGen3Module) tools (11, 12, 13). Since these tools use different knowledgebases and statistical methods, a more complete view of biological annotation is produced. There was generally a large overlap between the

results of the different annotation enrichment tools. Additionally, the different visualizations offered by each tool proved to be complementary.

Ingenuity Pathway Analysis (IPA):

The best gene sets for LV 5-Way and PBMC 5-Way datasets (as shown in Box 1 of main text) were analyzed using IPA. The analysis was performed using only the best gene sets, i.e., 75 genes for PBMC 5-Way, and 39 genes for LV 5-Way. A fold change cutoff of 1.0 was used for PBMC 5-Way, and cutoff of 1.5 was used for LV 5-Way during the analysis. The top enriched pathways were identified in each dataset on per pairwise comparison group basis. The top pathways for each pairwise comparison were sorted using p-value and organized into Tables S29 and S30. The dot plots (Figs. S10 and S11) were generated using the pathways and p-values from the tables, with pathways on the y-axis and pairwise comparisons on x-axis. The dots are color-coded by p-value significance, with blue dots representing lower significance and red representing higher significance.

Gene Set Enrichment Analysis (GSEAPreranked):

The GSEAPreranked analysis was performed using GSEA software version 4.2.3 with only the best gene sets identified during PBMC 5-Way (75 genes) and LV 5-Way (39 genes) analysis. The analysis struggled to attain significant p-values with such a small number of genes. The following gene set libraries were used: c2.reactome, c2.wikipathways, c2.kegg, c5.GO: biological processes, c8.all (cell type signatures) v 7.5.1. The required parameters were set as follows: number of permutations: 1000; minimum set size: 10; and maximum set size: 1000. The ranking metric used was log2(FC). Similar to IPA and Enrichr, the most significantly enriched pathways involved immune system and inflammation processes.

Blood Transcription Module (BTM) Analysis (BloodGen3Module):

In order to obtain a more complete annotation of the blood-based 5-Way PBMC best gene set, blood transcription module (BTM) analysis was performed using R BloodGen3Module version 1.4.0 package. Only the best gene set comprised of 75 genes from the PBMC 5-way analysis were input into the BloodGen3Module software. This analysis resulted in the differential module response status of 39 different BTM modules for each of the pairwise comparisons (Table S32). Cells in shades of red are upregulated for the condition listed first, and shades of green if downregulated for the condition listed first.

y. Codebase:

Github: https://github.com/staslist/Liver-Disease-Diagnostic The repository contains the code used to perform the analysis. Directories and sample names have been removed from the codebase.

2. Supplementary results

a. Best Gene Sets Fold Changes.

Listed below are fold changes corresponding to the best gene sets for LV 5-Way and PBMC 5-Way datasets as provided in the main text. The fold change (FC) is computed by taking the log2(q1/q2) wherein q1 is the first condition listed in the q1 v q2 format and q2 is the second listed condition. The bolded entries are significant according to false discovery rate (q-value) metric. For brevity only the pairwise comparisons involving controls (CT) are shown.

i. LV 5-Way.

Table S6: LV 5-Way best gene set directionality table.

	AH v CT		AC v CT		NF v CT		HP v CT	
	FC	Q-Value	FC	Q-Value	FC	Q-Value	FC	Q-Value
AKR1B10	**4.635**	9.89E-04	**2.15**	7.21E-03	**3.742**	9.89E-04	**3.008**	9.89E-04
ATF3	**-2.278**	9.89E-04	-1.147	1.74E-01	0.715	3.62E-01	0.297	7.30E-01
CYP2A6 (includes others)	**2.708**	7.91E-03	**3.206**	9.89E-04	**4.847**	9.89E-04	**4.463**	9.89E-04
CYP2B6	**-2.177**	9.89E-04	0.672	5.37E-01	1.343	8.08E-02	1.277	1.02E-01

DOCK7	**6.367**	9.89E-04	**4.327**	5.06E-03	0.444	7.21E-01	0.931	3.95E-01
DUSP1	**-1.86**	9.89E-04	0.455	5.95E-01	0.95	8.27E-02	**1.165**	3.41E-02
EPS8L1	**2.704**	9.89E-04	**3.202**	9.89E-04	**3.056**	9.89E-04	**3.355**	9.89E-04
GADD45B	**-2.835**	9.89E-04	-0.819	2.18E-01	0.292	6.80E-01	-0.052	9.56E-01
GADD45G	**-2.428**	9.89E-04	-0.559	4.28E-01	**1.182**	1.89E-03	**1.406**	2.73E-03
GSTA2	-2.796	2.92E-01	-0.485	7.60E-01	0.617	7.36E-01	0.983	5.86E-01
HBA1/HBA2	**3.437**	1.12E-02	0.104	9.20E-01	**2.493**	5.80E-03	**2.885**	9.89E-04
IFI27	1.09	5.80E-02	0.261	7.74E-01	0.992	1.12E-01	**4.941**	9.89E-04
IFI44L	1.097	6.34E-02	**2.944**	9.89E-04	-0.066	9.64E-01	**2.499**	9.89E-04
IFI6	0.628	2.45E-01	0.147	8.76E-01	0.534	4.65E-01	**4.075**	9.89E-04
IFITM1	**-5.535**	9.89E-04	**-5.466**	9.89E-04	**-6.084**	9.89E-04	-0.262	9.15E-01
IGFBP1	-0.793	2.02E-01	1.37	1.13E-01	**1.948**	2.73E-03	**2.047**	1.89E-03
IGHV3-23	0.492	6.21E-01	1.72	9.36E-02	-1.422	1.61E-01	0.666	4.98E-01
ISG15	0.695	1.55E-01	-0.398	5.87E-01	**1.365**	4.31E-03	**4.392**	9.89E-04
KRT23	**4.651**	9.89E-04	**3.702**	9.90E-03	**3.253**	8.59E-03	**4.026**	5.80E-03
KRT7	**2.401**	9.89E-04	**2.376**	9.89E-04	**3.173**	9.89E-04	**3.1**	9.89E-04
LINC01554	**-3.797**	9.89E-04	**-2.641**	9.89E-04	**-3.532**	9.89E-04	**-4.426**	9.89E-04
MMP7	**4.246**	9.89E-04	**3.479**	9.89E-04	**2.503**	9.89E-04	**2.085**	6.51E-03
MT1G	**-2.648**	9.89E-04	**-3.919**	2.73E-03	0.038	9.78E-01	-1.088	4.20E-01
MT1M	**-5.155**	9.89E-04	**-5.243**	9.89E-04	**-2.021**	1.89E-03	**-3.182**	9.89E-04
MUC1	0.214	9.05E-01	0.877	6.96E-01	**3.22**	2.44E-02	**3.126**	3.74E-02
MUC6	**2.099**	9.89E-04	**4.06**	9.89E-04	**3.782**	9.89E-04	**3.62**	9.89E-04
NR4A1	**-1.789**	4.96E-02	1.587	1.61E-01	1.882	7.04E-02	**2.16**	3.97E-02
OASL	**1.637**	2.00E-02	0.447	6.96E-01	1.235	1.52E-01	**3.753**	9.89E-04
PLA2G2A	**-5.022**	9.89E-04	**-4.848**	9.89E-04	**-2.545**	2.58E-02	-1.763	1.83E-01
PPP1R1A	**-4.325**	9.89E-04	**-3.138**	9.89E-04	**-1.577**	7.91E-03	**-2.89**	9.89E-04
RGS1	-0.885	2.53E-01	**2.52**	3.53E-03	**2.093**	3.53E-03	**3.005**	9.89E-04
S100A8	0.228	7.57E-01	**-3.269**	9.89E-04	-0.617	4.07E-01	-0.997	8.69E-02
SAA2-SAA4	1.961	5.58E-01	-1.113	7.18E-01	2.711	5.42E-01	0.727	8.02E-01
SCTR	**4.567**	9.89E-04	**3.488**	2.54E-02	**4.217**	9.89E-04	**4.326**	9.89E-04
SERHL2	1.435	2.42E-01	1.65	3.31E-01	**3.623**	4.31E-03	**2.8**	3.86E-02
SLC2A3	0.246	8.18E-01	1.056	2.71E-01	**2.805**	9.89E-04	**2.905**	9.89E-04
SPINK1	-2.443	2.28E-01	**-4.313**	9.89E-04	**-2.898**	9.89E-04	**-4.821**	9.89E-04
SYT8	1.626	1.51E-01	**4.065**	9.89E-04	**4.128**	9.89E-04	**4.443**	9.89E-04

*Green shading highlights positive fold change (up-regulation), and red shading highlights negative fold change (down-regulation). Bolded entries are significant according to q-value.

ii. PBMC 5-Way.

Table S7: PBMC 5-Way best gene set directionality table.

	AH v CT		DAAA v CT		NF v CT		HP v CT	
	FC	Q-Value	FC	Q-Value	FC	Q-Value	FC	Q-Value
AHSP	**6.398**	1.48E-03	**3.446**	1.48E-03	**1.557**	1.48E-03	**2.137**	1.48E-03
ALAS2	**4.577**	1.48E-03	**3.697**	1.48E-03	**1.394**	1.58E-02	**2.351**	1.48E-03
ALPL	**2.742**	1.48E-03	-0.042	9.85E-01	-0.889	1.37E-01	0.759	2.64E-01
ANXA3	**2.434**	1.48E-03	-0.325	6.26E-01	-0.019	9.92E-01	-0.773	6.73E-02

AQP9	**2.335**	1.48E-03	**1.2**	1.48E-03	0.487	7.49E-02	**1.211**	1.48E-03
ATF7IP2	**-0.874**	2.14E-02	-0.612	1.78E-01	-0.086	9.58E-01	**-1.093**	1.20E-02
AZU1	**1.49**	1.48E-03	-0.047	9.79E-01	0.368	6.16E-01	-0.658	3.03E-01
BCAT1	**2.263**	1.48E-03	**1.378**	1.48E-03	**1.017**	2.72E-03	**0.881**	3.19E-02
C1QA	**2.994**	1.48E-03	**1.403**	1.48E-03	0.214	8.04E-01	**1.094**	1.48E-03
C1QB	**3.697**	1.48E-03	**1.786**	1.48E-03	0.17	8.95E-01	**1.633**	1.48E-03
CAMP	**1.389**	1.48E-03	-0.344	4.89E-01	-0.006	9.97E-01	**-0.874**	2.53E-02
CCR2	**1.36**	1.48E-03	**1.064**	1.48E-03	-0.202	7.65E-01	0.557	8.00E-02
CD180	0.521	7.33E-02	**0.668**	8.64E-03	-0.428	2.83E-01	0.551	1.40E-01
CEACAM3	**1.964**	1.48E-03	0.725	6.81E-02	0.259	8.23E-01	**1.222**	1.48E-03
CEACAM8	**2.11**	1.12E-02	-0.117	9.64E-01	0.293	8.66E-01	-1.189	2.85E-01
CHI3L1	**2.149**	1.48E-03	0.217	8.03E-01	0.046	9.77E-01	-0.065	9.66E-01
CRISP3	**1.838**	1.48E-03	0.051	9.70E-01	0.478	3.48E-01	-1.051	5.35E-02
CTSG	**1.616**	1.48E-03	-0.266	8.24E-01	0.345	6.78E-01	-0.433	6.66E-01
CXCL5	**-1.636**	1.48E-03	**-1.559**	1.48E-03	-0.051	9.69E-01	**-0.818**	1.79E-02
CXCR1	**1.249**	1.48E-03	0.163	8.34E-01	-0.268	6.91E-01	**1.079**	1.48E-03
DEFA1 (includes others)	0.615	9.67E-02	**-1.274**	1.48E-03	**-0.72**	2.47E-02	**-1.728**	1.48E-03
DEFA4	**2.227**	1.48E-03	-0.469	4.98E-01	0.546	1.77E-01	-0.873	2.12E-01
DSC2	**2.649**	1.48E-03	**1.505**	1.48E-03	0.246	7.98E-01	0.093	9.54E-01
DYSF	**2.356**	1.48E-03	**1.278**	1.48E-03	0.443	2.18E-01	**1.04**	1.48E-03
ELANE	**2.347**	1.48E-03	-0.002	9.99E-01	0.457	4.76E-01	-0.51	5.09E-01
FCGR3A/FCGR3B	**0.804**	2.72E-03	-0.125	8.93E-01	-0.546	1.66E-01	**0.894**	5.87E-03
FFAR2	**1.161**	1.48E-03	0.322	5.51E-01	-0.25	7.68E-01	**1.199**	1.48E-03
FLVCR2	**2.292**	1.48E-03	**1.308**	1.48E-03	0.399	6.08E-01	0.871	5.30E-02
FPR2	**2.303**	1.48E-03	1.058	1.10E-01	0.018	9.95E-01	1.022	1.88E-01
GTF2IRD2/GTF2IRD2B	0.24	7.50E-01	0.547	1.75E-01	0.474	3.27E-01	**1.185**	6.82E-03
HBD	**6.483**	1.48E-03	**3.388**	1.48E-03	**1.76**	1.48E-03	**2.69**	1.48E-03
HBM	6.907	2.33E-01	**3.941**	1.48E-03	**1.618**	1.48E-03	**3.352**	1.48E-03
HBQ1	**3.467**	1.48E-03	**1.568**	1.48E-03	0.298	8.62E-01	0.834	2.18E-01
HP	**3.353**	1.48E-03	1.253	1.43E-01	0.642	5.93E-01	0.285	8.97E-01
IFITM3	**1.709**	1.48E-03	**0.679**	1.48E-03	-0.205	7.20E-01	**1.175**	1.48E-03
IGHG3	-0.749	1.19E-01	**-1.688**	1.48E-03	**-1.366**	1.48E-03	-0.813	5.97E-02
IGHG4	0.023	9.88E-01	**-0.857**	1.20E-02	**-1.307**	1.48E-03	-0.21	8.40E-01
IGKV1-12	0.587	2.73E-01	-0.389	4.86E-01	**-1.004**	2.27E-02	-0.032	9.85E-01
IGKV1-39	-0.292	7.39E-01	-0.609	1.84E-01	**-1.337**	1.48E-03	-0.615	2.33E-01
IGKV1D-13	-0.704	1.76E-01	**-1.402**	1.48E-03	-1.073	5.21E-02	-0.027	9.91E-01
IGLC3	-0.164	7.97E-01	**-0.793**	4.89E-03	**-1.085**	1.48E-03	-0.555	1.04E-01
IGLV3-10	-0.42	7.32E-01	-1.089	7.37E-02	**-1.624**	4.89E-03	-0.103	9.71E-01
KCNJ15	**1.617**	1.48E-03	0.585	1.44E-01	-0.036	9.85E-01	**1.331**	1.48E-03
LCN2	**2.224**	1.48E-03	-0.276	6.28E-01	0.235	7.44E-01	**-0.905**	2.53E-02
LTF	**2.084**	1.48E-03	0.06	9.61E-01	0.386	3.91E-01	-0.708	5.55E-02
MME	**1.325**	1.48E-03	**0.677**	3.31E-02	-0.168	8.83E-01	**1.107**	1.48E-03
MMP8	**3.867**	1.48E-03	0.199	7.34E-01	0.205	7.77E-01	**-1.043**	1.48E-03
MPO	**2.354**	1.48E-03	0.162	8.29E-01	0.526	1.65E-01	-0.43	3.09E-01

MPZL2	**0.754**	1.12E-02	**0.737**	1.94E-02	-0.337	6.70E-01	-0.199	8.61E-01
NLRC4	**1.635**	1.48E-03	**1.004**	1.48E-03	-0.195	8.44E-01	**0.648**	4.81E-02
NRP1	**1.329**	1.48E-03	**1.25**	1.48E-03	0.151	9.25E-01	0.403	6.59E-01
ORM1	**2.565**	1.48E-03	0.662	3.56E-01	0.547	5.99E-01	0.025	9.92E-01
OSBPL10	**-1.804**	1.48E-03	-0.495	2.20E-01	-0.132	9.09E-01	0.318	6.22E-01
PGLYRP1	**2.619**	1.48E-03	-0.234	9.18E-01	-0.013	9.97E-01	-0.64	7.21E-01
PLA2G4C	**0.916**	2.72E-03	0.553	1.32E-01	-0.265	7.49E-01	**1.12**	2.72E-03
PRRG4	**0.766**	1.03E-02	0.364	3.74E-01	-0.376	5.22E-01	-0.6	1.59E-01
PTK7	**-1.44**	1.48E-03	**-1.174**	1.48E-03	-0.372	6.15E-01	-0.971	1.20E-02
RAB10	**1.292**	1.48E-03	**0.919**	1.48E-03	0.029	9.83E-01	**0.997**	1.48E-03
RETN	**2.55**	1.48E-03	0.223	6.90E-01	-0.123	9.11E-01	0.645	6.37E-02
RNASE2	**2.366**	1.48E-03	**0.594**	2.59E-02	0.133	8.98E-01	0.458	2.57E-01
RNASE3	**1.712**	1.48E-03	-0.305	7.78E-01	0.447	4.88E-01	-0.669	4.08E-01
S100B	**-0.998**	3.65E-02	**-1.145**	2.07E-02	0.107	9.58E-01	0.409	5.49E-01
S100P	**2.345**	1.48E-03	0.138	9.16E-01	0.11	9.48E-01	0.864	1.15E-01
SC5D	-0.349	3.55E-01	-0.179	8.04E-01	0.324	5.39E-01	**-0.809**	8.64E-03
SIGLEC6	-0.576	2.70E-01	-0.094	9.52E-01	-0.446	5.19E-01	**1.223**	1.48E-03
SLC25A37	**3.529**	1.48E-03	**1.824**	1.48E-03	0.464	1.26E-01	**1.226**	8.64E-03
SLPI	**2.081**	1.48E-03	-0.535	3.89E-01	-0.027	9.91E-01	-0.384	7.13E-01
TCF7L2	**1.286**	1.48E-03	**1.274**	1.48E-03	**0.873**	1.48E-03	**1.365**	1.48E-03
TLR8	**1.305**	1.48E-03	**1.011**	1.48E-03	-0.109	8.94E-01	0.13	8.58E-01
TMEM144	**1.579**	1.48E-03	**1.126**	1.48E-03	-0.23	9.10E-01	0.081	9.69E-01
TMEM150B	**1.227**	1.48E-03	**0.88**	2.21E-02	-0.274	8.36E-01	0.442	5.57E-01
TMEM170B	**1.075**	1.48E-03	**0.508**	9.49E-03	0.206	6.10E-01	**-0.474**	4.08E-02
TNFSF10	**0.687**	1.48E-03	**0.785**	1.48E-03	-0.396	3.27E-01	0.588	6.69E-02
VSIG4	**2.567**	1.48E-03	0.27	6.97E-01	-0.321	7.05E-01	0.553	2.93E-01
ZNF683	**-1.473**	1.48E-03	-0.517	2.47E-01	-0.356	6.77E-01	0.76	7.45E-02

*Green shading highlights positive fold change (up-regulation), and red shading highlights negative fold change (down-regulation). Bolded entries are significant according to q-value.

b. Classification Performance, In Silico Biological Validation, and Top Enrichr Hits Tables.

Classification Performance Tables Description:

Listed below are the classification accuracies using nested cross-validation for our four datasets. For the LV 2-Way dataset, 36 configurations are given. For the 3 other datasets (LV 3-Way, LV 5-Way, and PBMC 5-Way), only the configuration that resulted in the best gene set are given. In the tables below, each dataset has a single entry highlighted in green and bolded. This denotes the configuration and feature size that produced the best gene set.

The classification performance tables are formatted as follows. The headings in the table indicate FS method (DE, IG) and Outlier Filter Threshold (2.5, 3.0, 3.5). Configurations are represented as: ML Classifier / Outlier Feature Filter Method.

Enrichr *In Silico* Biological Validation Tables Description:

The *in silico* biological validation tables contain the tallies that were attained via Enrichr for each dataset. Each of the 36 method configurations produced two gene sets, one attained via (4 out of 5) and (5 out of 5) gene set intersection, as described within Candidate Gene Set section of Supplemental Methods. The choice of the classifier did not impact the gene set generated. Therefore, there were only a total of 24 distinct gene set configurations (i.e., resulting from multiplying 2 FS methods (DE, IG) by 2 outlier filtering strategies (Intersection, Union) by 3 filter thresholds (2.5, 3.0, 3.5) by 2 merge strategies (4 out of 5 merge, and 5 out of 5 merge).

The configurations are listed above the tables: Outlier Filtering Strategy / Merge Strategy. The entries in the 1st column are: training set feature size (in outer loop of nested cross validation) – gene set size after merge for configuration #1/ #2/ … / #6. Cells containing “NA” indicate that the gene set size was zero after merge, and therefore, enrichment analysis could not be performed. The headings in the table indicate FS method (DE, IG) and Outlier Filter Standard Deviation Threshold (2.5, 3.0, 3.5). The values that are bolded and highlighted in green correspond to the best gene set for a given dataset. For all datasets, other than LV 2-Way, we only provided the configuration that resulted in the best gene set.

Top Enrichr Hits Tables Description:

The respective hits were sorted by adjusted p-value and filtered using the regular expression described in the methods, then the top 3 were selected for each category, for each one of the four datasets. Highly redundant entries (in either gene list or function) were removed.

i. LV 2-Way.

Classification Performance:

kNN / Intersection:

Table S8: The classification accuracies for kNN / Intersection configuration in LV 2-Way dataset.

Feature Size	DE 2.5	DE 3	DE 3.5	IG 2.5	IG 3	IG 3.5
2	0.79	0.82	0.82	0.94	0.92	0.92
3	0.92	0.86	0.81	0.97	0.95	0.86
4	0.97	0.97	0.93	0.97	1	0.97
5	0.97	0.95	0.97	0.97	0.97	0.97
10	0.97	0.97	0.97	0.97	0.97	0.95
15	0.97	0.97	0.97	0.97	0.97	0.97
20	0.97	0.97	0.97	0.97	0.97	0.97
25	0.97	0.97	0.97	0.97	0.97	0.97
50	0.97	0.97	0.97	0.97	0.97	0.97

kNN / Union:

Table S9: The classification accuracies for kNN / Union configuration in LV 2-Way dataset.

Feature Size	DE 2.5	DE 3	DE 3.5	IG 2.5	IG 3	IG 3.5
2	0.93	0.93	0.9	0.92	0.95	0.97
3	0.95	0.95	0.95	0.93	0.95	0.95
4	0.97	0.97	0.97	0.93	0.95	0.95
5	0.95	0.97	0.97	0.95	0.97	0.95
10	0.92	0.95	0.95	0.97	0.97	0.95
15	0.97	0.95	0.95	0.97	0.97	0.95
20	0.97	0.97	0.97	0.97	1	1
25	0.97	1	1	1	1	1
50	0.95	0.95	0.95	1	1	1

LR / Intersection:

Table S10: The classification accuracies for LR / Intersection configuration in LV 2-Way dataset.

Feature Size	DE 2.5	DE 3	DE 3.5	IG 2.5	IG 3	IG 3.5
2	0.82	0.82	0.82	0.87	0.86	0.92
3	0.95	0.9	0.77	0.93	0.88	0.92
4	0.92	0.88	0.88	0.95	0.95	0.92
5	0.92	0.97	0.97	0.95	0.95	0.97
10	0.97	0.97	0.97	0.97	0.97	0.97
15	0.97	0.97	0.97	0.97	0.97	0.97
20	0.97	0.97	0.97	0.97	0.97	0.97
25	0.97	0.97	0.97	0.97	0.97	0.97
50	1	1	1	0.97	0.97	0.97

LR / Union:

Table S11: The classification accuracies for LR / Union configuration in LV 2-Way dataset.

Feature Size	DE 2.5	DE 3	DE 3.5	IG 2.5	IG 3	IG 3.5
2	0.83	0.91	0.88	0.92	0.93	0.92
3	0.95	0.95	0.92	0.95	0.97	0.95
4	0.95	0.97	0.97	0.95	0.95	0.97
5	0.92	0.97	0.97	0.95	0.95	0.95
10	0.97	**0.97**	0.97	0.95	0.97	0.95
15	1	0.97	0.97	0.97	1	0.95
20	1	1	1	0.97	1	1
25	1	1	1	0.97	1	1
50	1	1	1	0.97	0.97	1

SVM / Intersection:

Table S12: The classification accuracies for SVM / Intersection configuration in LV 2-Way dataset.

Feature Size	DE 2.5	DE 3	DE 3.5	IG 2.5	IG 3	IG 3.5
2	0.82	0.82	0.82	0.93	0.92	0.97
3	0.87	0.86	0.74	0.97	0.9	0.97
4	0.95	0.95	0.95	0.97	0.95	0.95
5	0.93	0.92	0.9	0.97	0.95	0.95
10	0.95	0.95	0.95	1	0.97	0.97
15	0.95	0.95	0.95	0.97	0.97	0.97
20	0.95	0.95	0.95	0.97	0.97	0.97
25	0.97	0.95	0.95	0.97	0.97	0.97
50	1	0.97	0.97	0.97	0.97	0.97

SVM / Union:

Table S13: The classification accuracies for SVM / Union configuration in LV 2-Way dataset.

Feature Size	DE 2.5	DE 3	DE 3.5	IG 2.5	IG 3	IG 3.5
2	0.93	0.93	0.87	0.93	0.9	0.91
3	0.93	0.93	0.93	0.95	0.97	0.95
4	0.97	0.95	0.92	0.97	1	0.93
5	0.97	0.97	0.95	0.93	1	1
10	0.95	0.97	0.95	0.95	1	0.97
15	0.97	0.97	0.97	0.97	1	0.97
20	1	0.97	0.97	1	1	0.97
25	1	0.97	0.97	1	1	1
50	0.97	1	1	1	1	1

In Silico Biological Validation:

Intersection / 4 out of 5 Merge:

Table S14: The Enrichr hits Intersection / 4 out of 5 Merge configuration in LV 2-Way dataset.

	DE 2.5	DE 3	DE 3.5	IG 2.5	IG 3	IG 3.5
2 - 1/1/1/0/0/0	1/0/0	1/0/0	1/0/0	NA	NA	NA
3 - 2/2/1/0/0/0	16/0/12	16/0/12	1/0/0	NA	NA	NA
4 - 3/2/2/0/0/1	18/0/0	16/0/12	16/0/12	NA	NA	0/0/0

5 - 3/3/3/1/1/1	18/0/0	18/0/0	18/0/0	0/0/0	0/0/0	0/0/0
10 - 9/9/7/1/2/2	22/1/3	22/1/3	33/1/6	0/0/0	0/0/0	0/0/0
15 - 12/12/12/3/2/2	17/2/5	17/2/5	17/2/5	0/0/0	0/0/0	0/0/0
20 - 13/13/13/4/3/4	18/3/7	18/3/7	18/3/7	7/0/0	0/0/0	1/0/0
25 - 18/18/18/5/6/5	1/3/7	1/3/7	1/3/7	5/0/0	3/0/0	2/0/0
50 - 34/33/32/16/13/18	1/3/7	1/3/7	1/4/7	7/0/0	5/0/0	0/0/0

Intersection / 5 out of 5 Merge:

Table S15: The Enrichr hits Intersection / 5 out of 5 Merge configuration in LV 2-Way dataset.

	DE 2.5	DE 3	DE 3.5	IG 2.5	IG 3	IG 3.5
10 - 2/1/1/1/0/1	22/0/10	23/0/8	23/0/8	0/0/0	NA	0/0/0
15 - 6/6/5/2/0/1	32/2/6	32/2/6	30/1/4	0/0/0	NA	0/0/0
20 - 7/7/6/2/0/3	31/1/5	31/1/5	32/2/6	0/0/0	NA	1/0/0
25 - 8/8/7/2/0/3	32/1/4	32/1/4	31/1/5	0/0/0	NA	1/0/0
50 - 16/17/16/4/4/6	3/3/4	1/3/3	1/3/4	1/0/0	1/0/0	6/0/0

*Feature Sizes 2-5 resulted in gene sets of size 0 and were therefore excluded.

Union / 4 out of 5 Merge:

Table S16: The Enrichr hits Union / 4 out of 5 Merge configuration in LV 2-Way dataset.

	DE 2.5	DE 3	DE 3.5	IG 2.5	IG 3	IG 3.5
2 - 0/0/1/0/0/0	NA	NA	19/0/12	NA	NA	NA
3 - 0/0/1/0/0/0	NA	NA	19/0/12	NA	NA	NA
4 - 3/1/2/0/0/0	10/2/1	23/0/8	37/0/18	NA	NA	NA
5 - 3/3/3/0/0/0	10/2/1	30/2/4	44/0/5	NA	NA	NA
10 - 6/9/9/1/1/1	19/2/6	**28/3/6**	32/2/10	1/2/1	0/0/0	0/0/0
15 - 8/9/11/3/1/1/2	11/3/4	28/3/6	20/3/7	8/0/0	0/0/0	0/0/0
20 - 9/13/16/4/4/3	5/4/2	1/4/5	1/4/7	10/0/0	1/0/0	0/0/0

25 - 10/14/16/6/7/6	12/4/4	1/4/6	1/4/7	7/0/0	4/0/0	3/0/0
50 - 26/34/34/16/18/17	2/5/8	1/5/12	3/5/11	0/0/0	0/0/0	0/1/0

Union / 5 out of 5 Merge:

Table S17: The Enrichr hits Union / 5 out of 5 Merge configuration in LV 2-Way dataset.

	DE 2.5	DE 3	DE 3.5	IG 2.5	IG 3	IG 3.5
5 - 0/1/0/0/0/0	NA	23/0/8	NA	NA	NA	NA
10 – 1/4/4/0/0/0	4/0/5	32/1/4	32/1/4	NA	NA	NA
15 - 3/6/7/0/0/0	9/0/0	32/1/5	31/1/5	NA	NA	NA
20 - 5/7/7/2/0/0	15/2/1	34/1/4	31/1/5	2/1/1	NA	
25 - 5/8/10/3/0/1	15/2/1	32/2/4	13/3/3	2/0/1	NA	0/0/0
50 - 12/17/20/7/3/3	6/3/1	1/3/4	1/3/4	4/0/0	1/0/0	1/0/0

*Feature Sizes 2-4 resulted in gene sets of size 0 and were therefore excluded.

Top Enrichr Hits:

Table S18: The Enrichr top hits for LV 2-Way best gene set.

Pathway		
Term	Adjusted P-Value	Genes
Linoleic acid metabolism	0.00542024	AKR1B10;PLA2G2A
phospholipid metabolic process (GO:0006644)	0.0309956	PLA2G2A;FITM1
primary alcohol catabolic process (GO:0034310)	0.0309956	AKR1B10
Tissue		
HEPATOCYTE	1.88588e-05	AKR1B10;PPP1R1A;MT1M;PLA2G2A;SCTR;FITM1;KRT23;TREM2
LIVER (BULK TISSUE)	0.0212485	AKR1B10;MT1M;PLA2G2A;SCTR;FITM1
OMENTUM	0.0212485	MMP7;PPP1R1A;MT1M;PLA2G2A;TREM2
Disease		
Alcoholic Hepatitis DOID-12351 human GSE28619 sample 477	1.27297e-05	MMP7;AKR1B10;PLA2G2A;KRT23;TREM2

hepatocellular carcinoma DOID-684 human GSE57957 sample 660	0.00078694	MMP7;AKR1B10;MT1M;PLA2G2A
Carcinoma, Hepatocellular C0019204 human GSE6764 sample 407	0.00966049	AKR1B10;PLA2G2A;KRT23

ii. LV 3-Way.

Classification Performance:

kNN / Union:

Table S19: The classification accuracies for kNN / Union configuration in LV 3-Way dataset.

Feature Size	DE 2.5	DE 3	DE 3.5
10	0.82	0.86	0.83
25	0.85	0.86	0.83
50	0.86	0.85	0.9
100	0.88	0.9	0.85
150	0.88	0.9	**0.9**
200	0.9	0.9	0.9
250	0.88	0.9	0.91
300	0.88	0.88	0.9
350	0.88	0.88	0.9
400	0.9	0.88	0.88
450	0.9	0.9	0.88
500	0.9	0.9	0.9

Enrichr In Silico Biological Validation:

Union / 5 out of 5 Merge:

Table S20: The Enrichr hits Union / 5 out of 5 Merge configuration in LV 3-Way dataset.

Feature Size	DE 2.5	DE 3	DE 3.5
10 - 0/1/0	NA	23/0/8	NA
25 - 2/4/4	4/0/6	34/1/5	30/1/4
50 - 5/8/10	16/0/0	34/4/10	13/4/11
100 - 12/19/21	2/4/0	11/4/13	10/4/14

150 - 19/32/33	0/4/7	9/5/15	**13/5/17**
200 - 27/44/46	1/4/6	6/5/19	2/5/21
250 - 37/55/58	1/4/9	4/5/28	2/5/19
300 - 43/65/71	0/5/9	4/6/30	5/6/26
350 - 54/74/84	1/5/11	4/6/28	5/6/30
400 - 76/89/94	4/5/17	2/6/31	7/6/29
450 - 93/95/109	5/6/24	2/6/31	5/6/32
500 - 111/115/117	12/6/22	11/7/35	11/6/33

Top Enrichr Hits:

Table S21: The Enrichr top hits for LV 3-Way best gene set.

Pathway		
Term	Adjusted P-Value	Genes
Oncostatin M	0.0181233	CXCL6;AKR1B10;LCN2;HAMP;S100A8
IL-17 signaling pathway	0.022097	CXCL6;LCN2;S100A8
Endogenous Toll-like receptor signaling	0.0259474	VCAN;S100A8
Tissue		
HEPATOCYTE	2.53723e-07	FCN3;PLA2G2A;SCTR;FITM1;KRT23;TREM2;IGSF9;FAM198A;DBNDD1;CYP2A7;AKR1B10;CYP2B6;PPP1R1A;CREB3L3;LCN2;GPC3;MT1G;HAO2
LIVER (BULK TISSUE)	4.02437e-05	FCN3;PLA2G2A;SCTR;FITM1;IGSF9;FAM198A;CYP2A7;AKR1B10;CYP2B6;CREB3L3;GPC3;MT1G;HAMP;HAO2;CFTR
OMENTUM	0.00010671	CXCL6;FCN3;MMP7;PLA2G2A;TREM2;IGSF9;FAM198A;PPP1R1A;GPNMB;RGS1;GPC3;MT1G;EPS8L1;S100A8
Disease		
Alcoholic Hepatitis DOID-12351 human GSE28619 sample 477	8.50532e-09	CXCL6;VCAN;MMP7;AKR1B10;GPNMB;PLA2G2A;EEF1A2;LCN2;KRT23;TREM2
hepatocellular carcinoma DOID-684 human GSE39791 sample 663	4.53136e-05	CYP2A7;CXCL6;FCN3;MMP7;PPP1R1A;MT1G;HAMP;S100A8

Carcinoma, Hepatocellular C0019204 human GSE6764 sample 407	9.65272e-05	FCN3;CYP2B6;PPP1R1A;MT1G;HAMP;HAO2;S100A8

iii. LV 5-Way.

Classification Performance:

SVM / Intersection:

Table S22: The classification accuracies for SVM / Intersection configuration in LV 5-Way dataset.

Feature Size	DE 2.5	DE 3	DE 3.5
10	0.84	0.75	0.75
25	0.84	0.81	0.83
50	0.89	0.86	0.88
100	0.86	0.85	0.84
150	**0.91**	0.87	0.89
200	0.86	0.86	0.85
250	0.86	0.85	0.83
300	0.85	0.86	0.83
350	0.85	0.83	0.83
400	0.85	0.85	0.83
450	0.85	0.85	0.83
500	0.85	0.83	0.86

In Silico Biological Validation:

Intersection / 5 out of 5 Merge:

Table S23: The Enrichr hits Intersection / 5 out of 5 Merge configuration in LV 5-Way dataset.

Feature Size	DE 2.5	DE 3	DE 3.5
10 -3/1/1	17/0/4	19/0/12	19/0/12
25 -6/6/6	20/0/6	20/0/6	20/0/6
50 - 16/14/14	10/2/5	11/2/6	11/2/6
100 - 29/27/26	21/7/19	14/5/18	13/5/18

150 - 39/38/38	**25/7/27**	26/7/26	26/7/26
200 - 52/51/49	22/7/22	19/7/21	25/5/21
250 - 70/70/66	26/9/30	26/9/29	26/9/28
300 - 85/86/85	38/9/35	37/9/35	36/9/35
350 - 100/100/97	72/9/41	72/8/41	45/8/40
400 - 121/117/114	84/9/39	71/9/39	75/9/38
450 - 140/138/138	83/10/43	81/9/40	81/9/40
500 - 160/161/159	98/9/48	94/9/47	94/9/47

Top Enrichr Hits:

Table S24: The Enrichr top hits for LV 5-Way best gene set.

Pathway		
Term	Adjusted P-Value	Genes
Interferon alpha/beta signaling	2.59005e-05	IFITM1;IFI27;IFI6;ISG15;OASL
cytokine-mediated signaling pathway (GO:0019221)	0.00940058	IFITM1;MUC1;IFI27;GSTA2;IFI6;ISG15;OASL
Drug metabolism: cytochrome P450	0.0193383	CYP2A7;CYP2B6;GSTA2
Tissue		
LIVER (BULK TISSUE)	6.9614e-08	IGFBP1;IFITM1;SPINK1;GADD45B;PLA2G2A;MT1M;SCTR;KRT7;ISG15;SAA2-SAA4;IFI44L;OASL;CYP2A7;AKR1B10;CYP2B6;IFI27;GSTA2;MT1G;ATF3;MUC6
OMENTUM	6.9614e-08	IGFBP1;MMP7;GADD45B;DUSP1;PLA2G2A;MT1M;IGHV3-23;KRT7;HBA2;SAA2-SAA4;GADD45G;NR4A1;MUC1;IFI27;PPP1R1A;RGS1;MT1G;EPS8L1;S100A8;ATF3
HEPATOCYTE	3.61882e-07	IGFBP1;SPINK1;GADD45B;PLA2G2A;MT1M;SCTR;KRT7;KRT23;SAA2-SAA4;SYT8;GADD45G;CYP2A7;AKR1B10;CYP2B6;IFI27;PPP1R1A;GSTA2;MT1G;MUC6
Disease		

Alcoholic Hepatitis DOID-12351 human GSE28619 sample 477	1.07891e-08	IGFBP1;NR4A1;GADD45B;PPP1R1A;DUSP1;RGS1;MT1M;MT1G;IFI44L;ATF3;GADD45G
hepatocellular carcinoma DOID-684 human GSE39791 sample 663	3.37686e-07	IGFBP1;CYP2A7;IFITM1;MMP7;GADD45B;PPP1R1A;MT1M;MT1G;HBA2;S100A8
Carcinoma, Hepatocellular C0019204 human GSE6764 sample 407	4.39099e-07	IFITM1;SPINK1;AKR1B10;IFI27;PLA2G2A;IFI6;KRT23;ISG15;IFI44L

iv. PBMC 5-Way.

Classification Performance:

LR / Union:

Table S25: The classification accuracies for LR / Union configuration in PBMC 5-Way dataset.

Feature Size	DE 2.5	DE 3	DE 3.5
10	0.5	0.44	0.56
25	0.59	0.62	0.54
50	0.64	0.67	0.63
100	0.66	0.64	0.66
150	0.66	0.66	0.67
200	0.67	**0.75**	0.69
250	0.66	0.72	0.68
300	0.67	0.72	0.71
350	0.68	0.72	0.74
400	0.65	0.72	0.71
450	0.65	0.72	0.72
500	0.64	0.72	0.72

In Silico Biological Validation:

Union / 5 out of 5 Merge:

Table S26: The Enrichr hits Union / 5 out of 5 Merge configuration in PBMC 5-Way dataset.

Feature Size	DE 2.5	DE 3	DE 3.5
10 -1/1/3	0/1/0	0/0/2	10/0/1

25 -4/6/9	19/0/1	14/4/4	27/3/6
50 -6/13/18	25/0/3	27/6/3	24/4/7
100 - 13/36/39	28/0/4	35/7/11	26/7/7
150 - 16/51/67	3/0/9	33/7/15	35/9/18
200 - 22/75/89	6/2/14	**41/10/17**	49/11/21
250 - 25/91/122	28/4/15	48/10/22	44/12/27
300 - 31/109/148	18/3/9	47/10/21	60/12/30
350 - 35/131/168	15/3/10	40/10/17	69/12/26
400 - 39/153/191	12/4/10	37/8/18	78/12/32
450 - 45/170/213	19/1/16	33/9/18	72/13/31
500 - 52/192/240	13/0/13	56/10/21	73/14/29

Top Enrichr Hits:

Table S27: The Enrichr top hits for PBMC 5-Way best gene set.

Pathway		
Term	Adjusted P-Value	Genes
neutrophil mediated immunity (GO:0002446)	5.36008e-22	ORM1;CRISP3;FPR2;RETN;MPO;CXCL5;FCGR3B;CXCR1;CTSG;PGLYRP1;CAMP;ELANE;MME;ANXA3;DEFA4;AZU1;RNASE3;MMP8;RNASE2;CEACAM3;RAB10;SLPI;LCN2;CHI3L1;S100P;CEACAM8;LTF
Innate immune system	3.02077e-08	C1QB;C1QA;DEFA4;CD180;DEFA3;NLRC4;S100B;IGHG3;IGHG4;IGKV1-39;IGLC3;TLR8;CCR2
mucosal immune response (GO:0002385)	3.11393e-08	DEFA4;DEFA3;FFAR2;RNASE3;CAMP;LTF
Tissue		
PERIPHERAL BLOOD	7.95747e-24	ALAS2;ORM1;CRISP3;DYSF;HBD;FPR2;NLRC4;RETN;CXCL5;IGHG3;IGHG4;HBM;FCGR3B;CXCR1;IGKV1-12;TNFSF10;IGLC3;FLVCR2;FFAR2;AHSP;HBQ1;PGLYRP1;CAMP;CCR2;MPZL2;ZNF683;TMEM150B;MME;DEFA4;CD180;DEFA3;RNASE3;MMP8;TMEM170B;RNASE2;IGKV1D-13;CEACAM3;SLC25A37;SLPI;IGLV3-10;LCN2;ALPL;TLR8;CHI3L1;S100P;SIGLEC6;LTF
GRANULOCYTE	8.46854e-12	ORM1;CRISP3;DYSF;FPR2;NLRC4;PRRG4;FCGR3B;CXCR1;TNFSF10;FFAR2;VSIG4;PGLYRP1;CAMP;ELAN

		E;MPZL2;MME;ANXA3;DEFA4;KCNJ15;DEFA3;RNASE3;MMP8;TMEM170B;RNASE2;CEACAM3;SLC25A37;SLPI;LCN2;ALPL;TLR8;CHI3L1;S100P;CEACAM8;LTF
WholeBlood	8.32759e-06	CEACAM3;FCGR3B;CXCR1;AQP9;KCNJ15;DYSF;TNFSF10;ALPL;TLR8;CHI3L1;FFAR2;FPR2
Disease		
Septic Shock C0036983 human GSE9692 sample 307	1.67821e-26	C1QB;C1QA;ORM1;CRISP3;AQP9;HP;DYSF;FPR2;NLRC4;RETN;MPO;CXCR1;FFAR2;VSIG4;PGLYRP1;CCR2;ANXA3;DEFA4;KCNJ15;RNASE3;MMP8;RNASE2;SLPI;LCN2;ALPL;TLR8;S100P;CEACAM8;BCAT1;LTF
familial combined hyperlipidemia DOID-13809 human GSE11393 sample 773	2.72273e-21	IFITM3;MME;ANXA3;DEFA4;AQP9;DYSF;DEFA3;FPR2;RNASE3;MMP8;RNASE2;CEACAM3;SLC25A37;FCGR3B;CXCR1;LCN2;ALPL;CHI3L1;S100P;FFAR2;CEACAM8;CAMP;LTF
hepatitis C virus related hepatocellular carcinoma UMLS CUI-C1333978 human GSE58208 sample 736	3.48243e-08	DEFA4;DYSF;DEFA3;HBD;MPO;HBM;SLPI;CXCR1;LCN2;S100P;FFAR2;AHSP;CEACAM8;LTF

c. Per Replicate RNA-seq Count Heatmaps:

Per replicate RNA-seq count heatmaps are provided to visualize the gene expression counts for each individual sample. The heatmaps are displayed using the best gene sets shown in Box 1 of the main text. In the figures below, the first heatmaps display the gene expression in our data, and the second heatmaps display the gene expression in the independent test dataset. For PBMC 5-Way dataset, only the heatmap of our data is provided.

i. LV 2-Way.

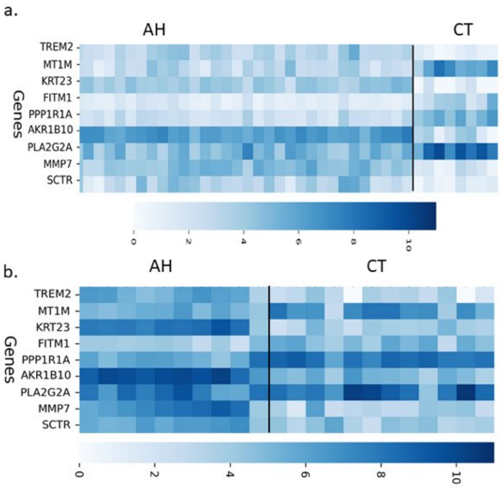

Fig. S6: LV 2-Way; Per Replicate Heatmap of Counts for Best Gene Set. a. Per replicate heatmap of best LV 2-Way gene set. b. Per replicate heatmap of best gene set within validation dataset.

ii. LV 3-Way.

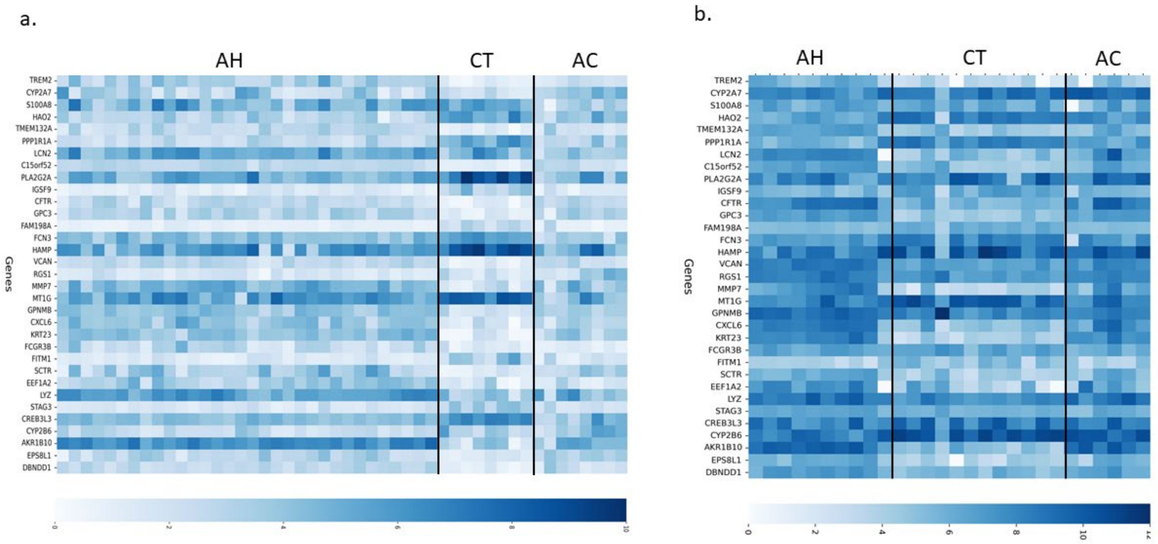

Fig. S7: LV 3-Way; Per Replicate Heatmap of Counts for Best Gene Set. a. Per replicate heatmap of best LV 3-Way gene set. b. Per replicate heatmap of best gene set within validation dataset.

iii. LV 5-Way.

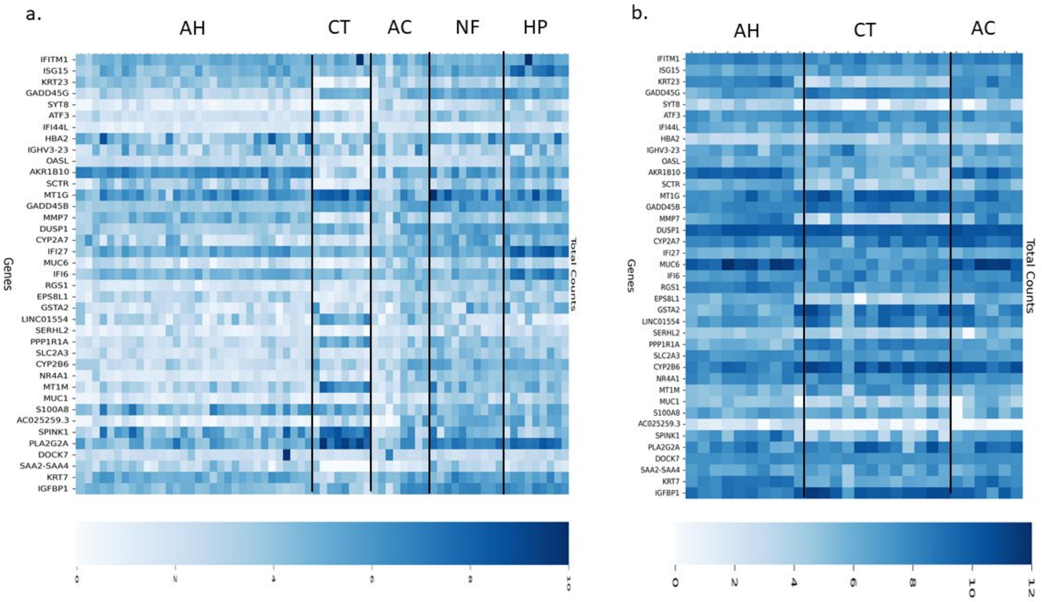

Fig. S8: LV 5-Way; Per Replicate Heatmap of Counts for Best Gene Set. a. Per replicate heatmap of best LV 5-Way gene set. b. Per replicate heatmap of best gene set within validation dataset.

iv. PBMC 5-Way.

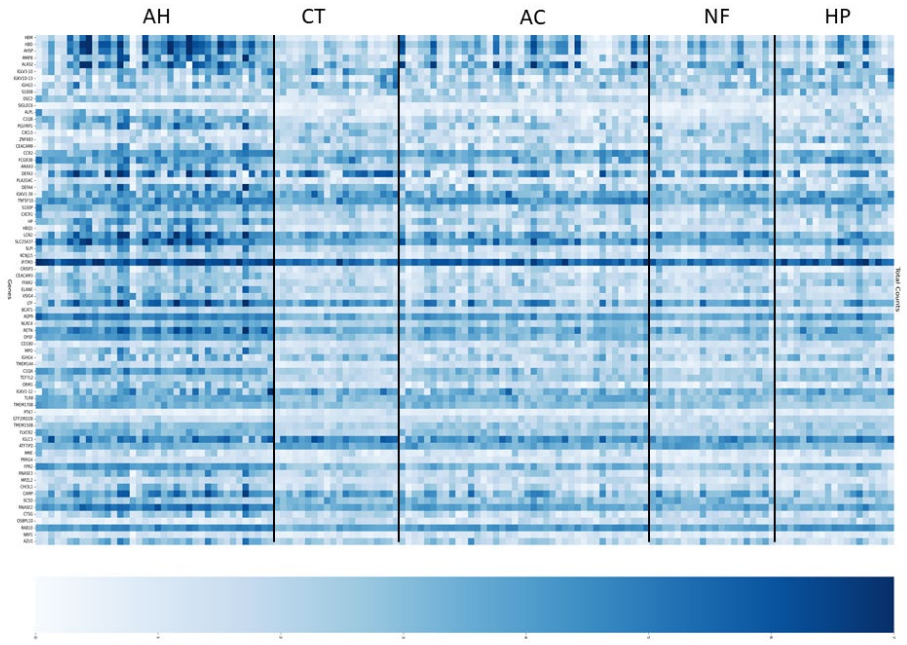

Fig. S9: PBMC 5-Way; Per replicate heatmap of best PBMC 5-Way gene set.

d. Comparison of additional in silico biological validation approaches:

i. IPA.

1. LV 5-Way:

Table S28: Top enriched IPA pathways per pairwise comparison in LV 5-Way best gene set.

Ingenuity Canonical Pathways	Pairwise Comparison	p-value	Molecules
PXR/RXR Activation	AH vs AC	1.10E-05	CYP2B6,GSTA2,IGFBP1
Acetone Degradation I (to Methylglyoxal)	AH vs AC	2.29E-04	AKR1B10,CYP2B6
Estrogen Biosynthesis	AH vs AC	3.89E-04	AKR1B10,CYP2B6
GADD45 Signaling	AH vs AC	7.76E-04	GADD45B,GADD45G
Senescence Pathway	AH vs AC	1.05E-03	GADD45B,GADD45G,SAA2-SAA4
SPINK1 General Cancer Pathway	CT vs AC	1.78E-05	MT1G,MT1M,SPINK1
Acetone Degradation I (to Methylglyoxal)	CT vs AC	3.31E-04	AKR1B10,CYP2A6 (includes others)
Estrogen Biosynthesis	CT vs AC	5.75E-04	AKR1B10,CYP2A6 (includes others)
Atherosclerosis Signaling	CT vs AC	5.01E-03	PLA2G2A,S100A8
Glucocorticoid Receptor Signaling	CT vs AC	1.00E-02	KRT23,KRT7,PLA2G2A
Acetone Degradation I (to Methylglyoxal)	CT vs AH	4.68E-06	AKR1B10,CYP2A6 (includes others),CYP2B6
Estrogen Biosynthesis	CT vs AH	1.07E-05	AKR1B10,CYP2A6 (includes others),CYP2B6
Bupropion Degradation	CT vs AH	2.75E-04	CYP2A6 (includes others),CYP2B6
Oxidative Ethanol Degradation III	CT vs AH	4.07E-04	CYP2A6 (includes others),CYP2B6
Nicotine Degradation III	CT vs AH	1.20E-03	CYP2A6 (includes others),CYP2B6
Interferon Signaling	HP vs AC	7.24E-07	IFI6,IFITM1,ISG15
Antioxidant Action of Vitamin C	HP vs AC	1.35E-03	PLA2G2A,SLC2A3
Atherosclerosis Signaling	HP vs AC	1.86E-03	PLA2G2A,S100A8
Role of IL-17A in Psoriasis	HP vs AC	7.24E-03	S100A8
Vitamin-C Transport	HP vs AC	1.17E-02	SLC2A3
Interferon Signaling	HP vs AH	7.76E-06	IFI6,IFITM1,ISG15
PXR/RXR Activation	HP vs AH	4.17E-05	CYP2B6,GSTA2,IGFBP1
Acetone Degradation I (to Methylglyoxal)	HP vs AH	5.37E-04	AKR1B10,CYP2B6
Estrogen Biosynthesis	HP vs AH	9.33E-04	AKR1B10,CYP2B6
GADD45 Signaling	HP vs AH	1.82E-03	GADD45B,GADD45G

Acetone Degradation I (to Methylglyoxal)	HP vs CT	5.37E-04	AKR1B10,CYP2A6 (includes others)
Interferon Signaling	HP vs CT	6.92E-04	IFI6,ISG15
Estrogen Biosynthesis	HP vs CT	9.33E-04	AKR1B10,CYP2A6 (includes others)
SPINK1 General Cancer Pathway	HP vs CT	1.95E-03	MT1M,SPINK1
PXR/RXR Activation	HP vs CT	2.09E-03	CYP2A6 (includes others),IGFBP1
SPINK1 General Cancer Pathway	NF vs AC	6.17E-04	MT1G,MT1M
Senescence Pathway	NF vs AC	6.76E-04	ATF3,GADD45G,SAA2-SAA4
Antioxidant Action of Vitamin C	NF vs AC	1.86E-03	PLA2G2A,SLC2A3
Atherosclerosis Signaling	NF vs AC	2.57E-03	PLA2G2A,S100A8
Role of IL-17A in Psoriasis	NF vs AC	8.51E-03	S100A8
PXR/RXR Activation	NF vs AH	4.90E-07	CYP2A6 (includes others),CYP2B6,GSTA2,IGFBP1
Bupropion Degradation	NF vs AH	2.75E-04	CYP2A6 (includes others),CYP2B6
Oxidative Ethanol Degradation III	NF vs AH	4.07E-04	CYP2A6 (includes others),CYP2B6
Acetone Degradation I (to Methylglyoxal)	NF vs AH	5.01E-04	CYP2A6 (includes others),CYP2B6
Estrogen Biosynthesis	NF vs AH	8.51E-04	CYP2A6 (includes others),CYP2B6
Acetone Degradation I (to Methylglyoxal)	NF vs CT	3.72E-04	AKR1B10,CYP2A6 (includes others)
Estrogen Biosynthesis	NF vs CT	6.31E-04	AKR1B10,CYP2A6 (includes others)
SPINK1 General Cancer Pathway	NF vs CT	1.35E-03	MT1M,SPINK1
PXR/RXR Activation	NF vs CT	1.45E-03	CYP2A6 (includes others),IGFBP1
Antioxidant Action of Vitamin C	NF vs CT	4.07E-03	PLA2G2A,SLC2A3
Interferon Signaling	HP vs NF	2.45E-07	IFI6,IFITM1,ISG15
Systemic Lupus Erythematosus In B Cell Signaling Pathway	HP vs NF	1.15E-02	IGHV3-23,ISG15
Coronavirus Replication Pathway	HP vs NF	1.58E-02	IFITM1
SPINK1 Pancreatic Cancer Pathway	HP vs NF	2.04E-02	SPINK1
SPINK1 General Cancer Pathway	HP vs NF	2.29E-02	SPINK1

* The colors are alternated between blue and white to highlight each pairwise comparison group.

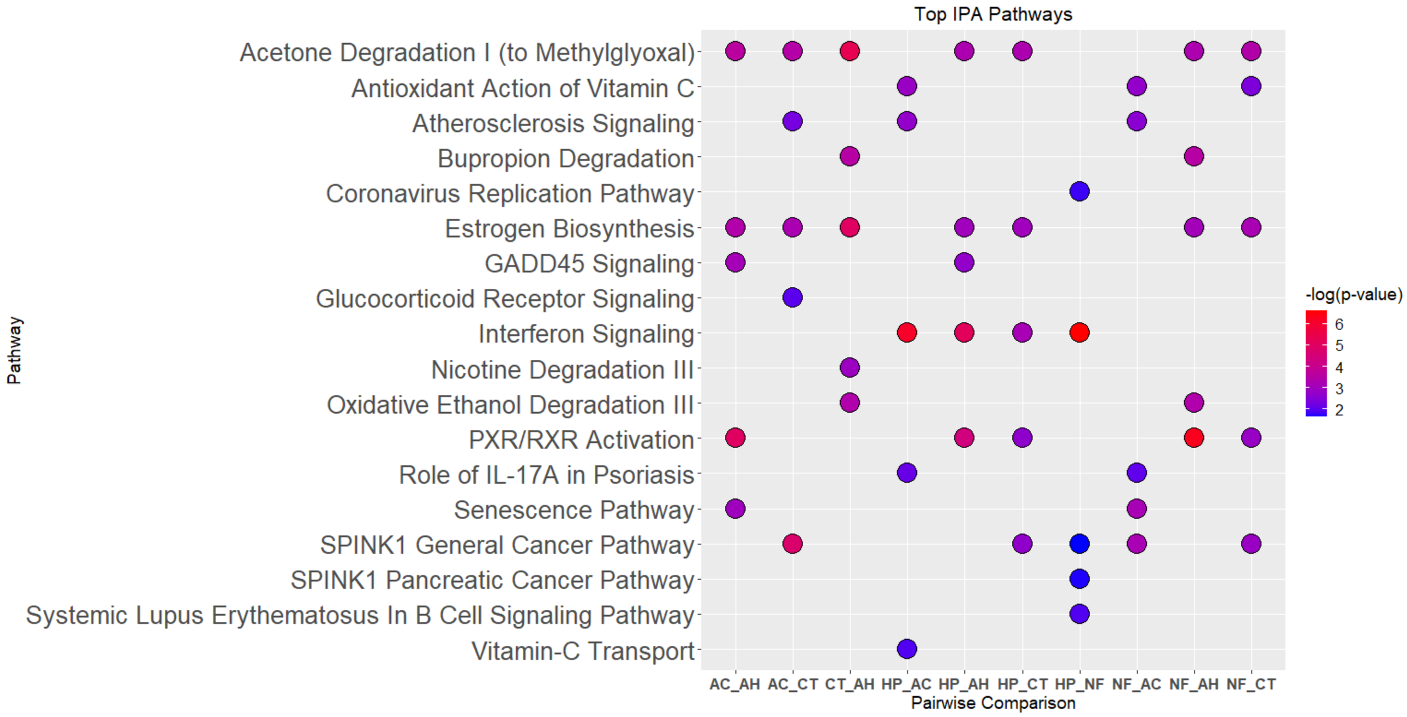

Fig. S10: Dot plot of top 5 IPA pathways and their p-value significance for each pairwise comparison of LV 5-Way best gene set. The dots are color-coded by p-value significance, with blue dots representing lower significance and red representing higher significance.

2. *PBMC 5-Way.*

Table S29: Top enriched IPA pathways per pairwise comparison in PBMC 5-Way best gene set.

Ingenuity Canonical Pathways	Pairwise Comparison	-log(p-value)	Molecules
Airway Pathology in Chronic Obstructive Pulmonary Disease	CT vs AH	4.07E-07	CTSG,ELANE,LCN2,MMP8,MPO,ORM1
Iron homeostasis signaling pathway	CT vs AH	2.19E-05	ALAS2,HBD,HBQ1,HP,SLC25A37
Granulocyte Adhesion and Diapedesis	CT vs AH	7.94E-05	CCR2,CXCL5,CXCR1,FPR2,MMP8
Airway Inflammation in Asthma	CT vs AH	7.94E-05	ELANE,RNASE2,RNASE3
Role of Pattern Recognition Receptors in Recognition of Bacteria and Viruses	CT vs AH	5.13E-04	C1QA,C1QB,NLRC4,TLR8
Airway Pathology in Chronic Obstructive Pulmonary Disease	AC vs AH	5.50E-08	CTSG,ELANE,LCN2,MMP8,MPO,ORM1
Airway Inflammation in Asthma	AC vs AH	3.02E-05	ELANE,RNASE2,RNASE3
Iron homeostasis signaling pathway	AC vs AH	1.00E-04	HBD,HBQ1,HP,SLC25A37

Acute Phase Response Signaling	AC vs AH	3.55E-04	C1QA,C1QB,HP,ORM1
IL-8 Signaling	AC vs AH	5.37E-04	AZU1,CXCR1,DEFA1 (includes others),MPO
Iron homeostasis signaling pathway	AC vs CT	1.66E-05	ALAS2,HBD,HBQ1,SLC25A37
Role of Pattern Recognition Receptors in Recognition of Bacteria and Viruses	AC vs CT	2.40E-05	C1QA,C1QB,NLRC4,TLR8
Complement System	AC vs CT	8.13E-04	C1QA,C1QB
TREM1 Signaling	AC vs CT	3.24E-03	NLRC4,TLR8
Pyroptosis Signaling Pathway	AC vs CT	5.01E-03	NLRC4,TLR8
Airway Pathology in Chronic Obstructive Pulmonary Disease	HP vs AH	1.02E-07	CTSG,ELANE,LCN2,MMP8,MPO,ORM1
Iron homeostasis signaling pathway	HP vs AH	7.08E-06	ALAS2,HBD,HBQ1,HP,SLC25A37
Airway Inflammation in Asthma	HP vs AH	3.98E-05	ELANE,RNASE2,RNASE3
Acute Phase Response Signaling	HP vs AH	5.13E-04	C1QA,C1QB,HP,ORM1
Melatonin Degradation III	HP vs AH	2.04E-03	MPO
Iron homeostasis signaling pathway	HP vs CT	3.39E-04	ALAS2,HBD,SLC25A37
Complement System	HP vs CT	6.31E-04	C1QA,C1QB
Tetrapyrrole Biosynthesis II	HP vs CT	5.13E-03	ALAS2
Heme Biosynthesis II	HP vs CT	9.33E-03	ALAS2
Role of Pattern Recognition Receptors in Recognition of Bacteria and Viruses	HP vs CT	9.77E-03	C1QA,C1QB
Tetrapyrrole Biosynthesis II	HP vs AC	2.57E-03	ALAS2
Heme Biosynthesis II	HP vs AC	4.68E-03	ALAS2
Inhibition of Matrix Metalloproteases	HP vs AC	1.95E-02	MMP8
FcÎ³ Receptor-mediated Phagocytosis in Macrophages and Monocytes	HP vs AC	4.68E-02	FCGR3A/FCGR3B
Airway Pathology in Chronic Obstructive Pulmonary Disease	HP vs AC	5.75E-02	MMP8
Phagosome Formation	HP vs NF	3.98E-04	CXCR1,FCGR3A/FCGR3B,FFAR2,IGHG4,PLA2G4C
Airway Pathology in Chronic Obstructive Pulmonary Disease	HP vs NF	5.50E-03	LCN2,MMP8
Granulocyte Adhesion and Diapedesis	HP vs NF	1.26E-02	CXCR1,MMP8
Cholesterol Biosynthesis I	HP vs NF	1.29E-02	SC5D
Cholesterol Biosynthesis II (via 24,25-dihydrolanosterol)	HP vs NF	1.29E-02	SC5D
Airway Pathology in Chronic Obstructive Pulmonary Disease	NF vs AH	4.27E-08	CTSG,ELANE,LCN2,MMP8,MPO,ORM1,TNFSF10

Iron homeostasis signaling pathway	NF vs AH	4.68E-05	ALAS2,HBD,HBQ1,HP,SLC25A37
Role of Pattern Recognition Receptors in Recognition of Bacteria and Viruses	NF vs AH	7.24E-05	C1QA,C1QB,NLRC4,TLR8,TNFSF10
Airway Inflammation in Asthma	NF vs AH	1.26E-04	ELANE,RNASE2,RNASE3
Granulocyte Adhesion and Diapedesis	NF vs AH	1.66E-04	CCR2,CXCL5,CXCR1,FPR2,MMP8
IL-15 Signaling	NF vs CT	1.55E-09	IGHG3,IGHG4,IGKV1-12,IGKV1-39,IGLC3,IGLV3-10
B Cell Receptor Signaling	NF vs CT	1.07E-08	IGHG3,IGHG4,IGKV1-12,IGKV1-39,IGLC3,IGLV3-10
Systemic Lupus Erythematosus In B Cell Signaling Pathway	NF vs CT	3.63E-08	IGHG3,IGHG4,IGKV1-12,IGKV1-39,IGLC3,IGLV3-10
Communication between Innate and Adaptive Immune Cells	NF vs CT	4.27E-08	IGHG3,IGHG4,IGKV1-12,IGKV1-39,IGLC3,IGLV3-10
Primary Immunodeficiency Signaling	NF vs CT	2.00E-06	IGHG3,IGHG4,IGLC3
Role of Pattern Recognition Receptors in Recognition of Bacteria and Viruses	NF vs AC	2.04E-07	C1QA,C1QB,NLRC4,TLR8,TNFSF10
Iron homeostasis signaling pathway	NF vs AC	6.46E-06	ALAS2,HBD,HBQ1,SLC25A37
Erythropoietin Signaling Pathway	NF vs AC	5.01E-04	HBD,HBQ1,TNFSF10
Complement System	NF vs AC	5.13E-04	C1QA,C1QB
TREM1 Signaling	NF vs AC	2.04E-03	NLRC4,TLR8

* The colors are alternated between blue and white to highlight each pairwise comparison group.

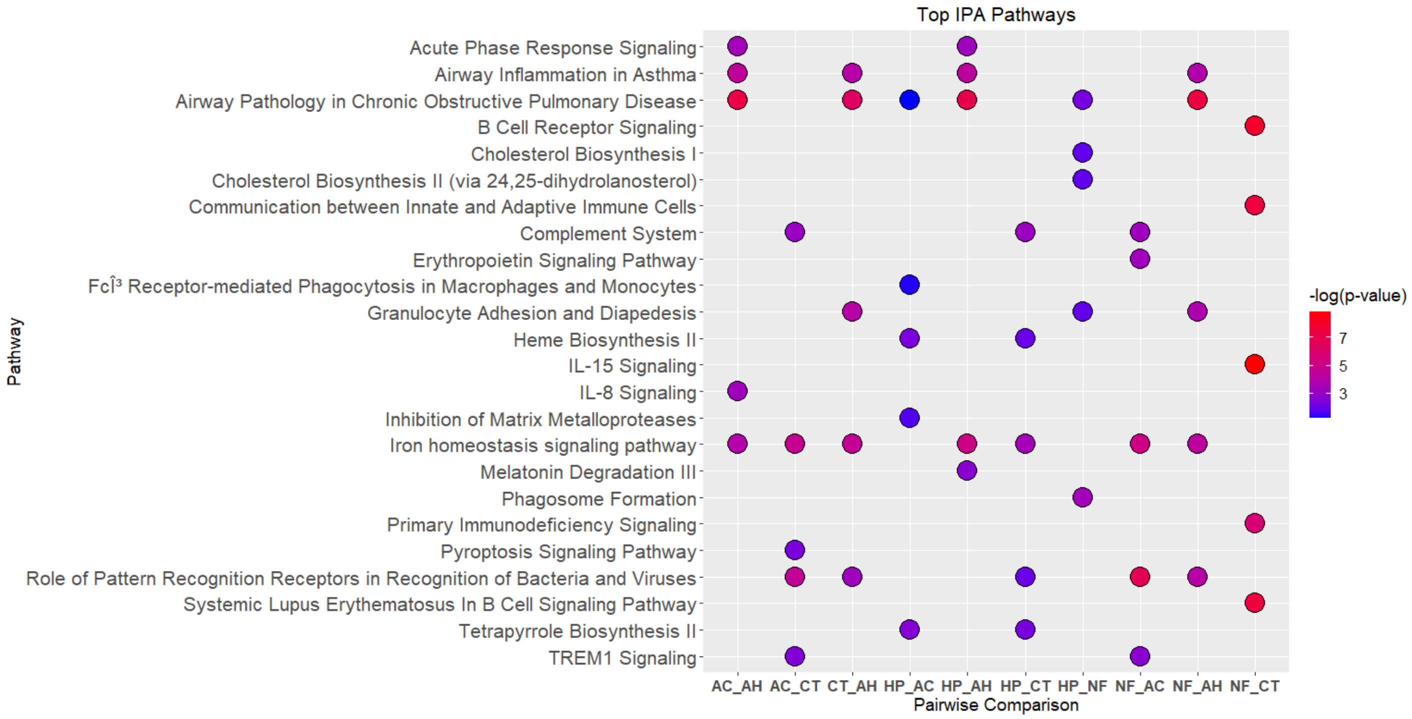

Fig. S11: Dot plot of top 5 IPA pathways and their p-value significance for each pairwise comparison of PBMC 5-Way best gene set. The dots are color-coded by p-value significance, with blue dots representing lower significance and red representing higher significance.

ii. GSEAPreranked.

1. LV 5-Way.

Table S30: Top enriched GSEA pathways per pairwise comparison in LV 5-Way best gene set.

GSEA Canonical Pathways	Pairwise Comparison	NES	p-value
Biological process involved in interspecies interaction between organisms	AH vs CT	1.374	0.098
Homeostatic process	AH vs CT	1.010	0.464
Regulation of intracellular signal transduction	AH vs CT	0.980	0.478
Pancreas ductal cell	AH vs CT	-1.178	0.255
Pancreas ductal cell	AH vs AC	1.193	0.247
Biological process involved in interspecies interaction between organisms	AH vs AC	0.988	0.473
Homeostatic process	AH vs AC	-1.527	0.047
Regulation of intracellular signal transduction	AH vs AC	-0.681	0.879
Homeostatic process	AH vs NF	-0.848	0.747
Regulation of intracellular signal transduction	AH vs NF	1.273	0.167

Pancreas ductal cell	AH vs NF	1.052	0.418
Biological process involved in interspecies interaction between organisms	AH vs NF	0.491	0.996
Homeostatic process	AH vs HP	-0.938	0.545
Biological process involved in interspecies interaction between organisms	AH vs HP	1.767	0.002
Regulation of intracellular signal transduction	AH vs HP	0.972	0.503
Pancreas ductal cell	AH vs HP	0.912	0.595
Homeostatic process	CT vs AC	-1.529	0.057
Biological process involved in interspecies interaction between organisms	CT vs AC	-1.272	0.182
Regulation of intracellular signal transduction	CT vs AC	-1.105	0.315
Pancreas ductal cell	CT vs AC	1.532	0.063
Pancreas ductal cell	CT vs NF	1.457	0.053
Homeostatic process	CT vs NF	0.851	0.654
Regulation of intracellular signal transduction	CT vs NF	0.539	0.968
Biological process involved in interspecies interaction between organisms	CT vs NF	-1.655	0.009
Pancreas ductal cell	CT vs HP	1.349	0.120
Biological process involved in interspecies interaction between organisms	CT vs HP	1.170	0.287
Homeostatic process	CT vs HP	0.921	0.579
Regulation of intracellular signal transduction	CT vs HP	0.783	0.730
Regulation of intracellular signal transduction	AC vs NF	1.227	0.229
Homeostatic process	AC vs NF	1.147	0.285
Pancreas ductal cell	AC vs NF	0.660	0.876
Biological process involved in interspecies interaction between organisms	AC vs NF	-1.030	0.412
Pancreas ductal cell	AC vs HP	-0.953	0.492
Biological process involved in interspecies interaction between organisms	AC vs HP	1.848	0.001
Homeostatic process	AC vs HP	1.363	0.104
Regulation of intracellular signal transduction	AC vs HP	1.100	0.366
Biological process involved in interspecies interaction between organisms	NF vs HP	1.848	0.001
Homeostatic process	NF vs HP	1.363	0.104
Regulation of intracellular signal transduction	NF vs HP	1.100	0.366
Pancreas ductal cell	NF vs HP	-0.953	0.492

* The colors are alternated between blue and white to highlight each pairwise comparison group. NES is the Normalized Enrichment Score calculated by GSEA.

2. *PBMC 5-Way.*

Table S31: Top enriched GSEA pathways per pairwise comparison in PBMC 5-Way best gene set.

GSEA Canonical Pathways	Pairwise Comparison	NES	p-value
Neutrophil degranulation	AH vs CT	-1.444	0.051
Defense response to bacterium	AH vs CT	-1.337	0.123
Innate immune system	AH vs CT	-1.279	0.144
Cell cell signaling	AH vs CT	1.319	0.130
Cellular response to oxygen containing compound	AH vs CT	1.197	0.238
Locomotion	AH vs CT	1.075	0.347
Neutrophil degranulation	AH vs AC	-1.829	0
Innate immune system	AH vs AC	-1.733	0.001
Defense response	AH vs AC	-1.692	0.001
Biological process involved in interspecies interaction between organisms	AH vs AC	-1.612	0.007
Response to bacterium	AH vs AC	-1.593	0.004
Response to molecule of bacterial origin	AH vs AC	-1.592	0.005
Lung proliferating macrophage cell	AH vs NF	-1.510	0.054
Pancreas ductal cell	AH vs NF	-1.484	0.062
Lung neutrophil cell	AH vs NF	-1.437	0.085
Innate immune system	AH vs NF	-1.434	0.073
Neutrophil degranulation	AH vs NF	-1.424	0.073
Homeostatic process	AH vs NF	-1.336	0.140
Neutrophil degranulation	AH vs HP	-1.783	0
Defense response	AH vs HP	-1.697	0.001
Defense response to bacterium	AH vs HP	-1.695	0.002
Response to bacterium	AH vs HP	-1.689	0.002
Antimicrobial humoral response	AH vs HP	-1.577	0.007
Innate immune system	AH vs HP	-1.571	0.010
Antimicrobial humoral response	CT vs AC	-2.049	0
Response to lipid	CT vs AC	-1.908	0
Response to molecule of bacterial origin	CT vs AC	-1.785	0.014
Response to bacterium	CT vs AC	-1.734	0.017
Chemical homeostasis	CT vs AC	1.600	0.015
Homeostatic process	CT vs AC	1.507	0.022
Adaptive immune response	CT vs NF	-2.229	0
Immune response	CT vs NF	-1.800	0
Vesicle mediated transport	CT vs NF	-1.532	0.055
Leukocyte mediated immunity	CT vs NF	-1.527	0.045
Chemical homeostasis	CT vs NF	1.443	0.068

Neutrophil degranulation	CT vs NF	1.433	0.058
Defense response to bacterium	CT vs HP	-2.518	0
Response to bacterium	CT vs HP	-2.449	0
Antimicrobial humoral response	CT vs HP	-2.421	0
Antimicrobial peptides	CT vs HP	-2.238	0
Response to molecule of bacterial origin	CT vs HP	-1.934	0.003
Response to lipid	CT vs HP	-1.928	0.004
Antimicrobial humoral response	AC vs NF	2.590	0
Defense response to bacterium	AC vs NF	2.037	0
Response to lipid	AC vs NF	2.009	0
Response to molecule of bacterial origin	AC vs NF	1.973	0.005
Response to bacterium	AC vs NF	1.882	0
Antimicrobial peptides	AC vs NF	1.822	0.013
Phosphorylation	AC vs HP	-1.540	0.067
Antimicrobial peptides	AC vs HP	-1.512	0.045
Positive regulation of molecular function	AC vs HP	-1.421	0.101
Programmed cell death	AC vs HP	-1.419	0.108
Lung neutrophil cell	AC vs HP	1.999	0.004
Adaptive immune response	AC vs HP	1.530	0.038
Lung neutrophil cell	NF vs HP	1.898	0.004
Adaptive immune response	NF vs HP	1.564	0.037
Antimicrobial humoral response	NF vs HP	-2.647	0
Antimicrobial peptides	NF vs HP	-2.472	0
Defense response to bacterium	NF vs HP	-2.323	0
Neutrophil degranulation	NF vs HP	-2.140	0

* The colors are alternated between blue and white to highlight each pairwise comparison group. NES is the Normalized Enrichment Score calculated by GSEA.

iii. Blood Transcription Module analysis (BloodGen3Module).

1. PBMC 5-Way.

Differential blood transcription module analysis was performed on our best gene set from the 5-way PBMC dataset, using BloodGen3Module software. Thirty-nine modules were identified as shown in Table S32. The cells of the table are color-coded according to the direction and expression level of the module for each comparison group. The AH group demonstrated upregulation in most modules. Differential blood transcription module analysis is useful for adding annotation to PBMC gene expression research (14, 15).

Table S32: Blood Transcription Module (BTM) response by pairwise comparison of PBMC 5-Way best gene set.

Module name	AH_CT	AH_AC	AH_NF	AH_HP	AC_CT	AC_NF	AC_HP	NF_CT	HP_CT	HP_NF	Module title	Top GOTERM BP
M13.18											B cells	RNA processing
M12.8											B cells	B cell activation
M16.107											Oxidative stress	N/A
M9.1											Cytotoxic lymphocytes	cellular defense response
M14.27											Protein synthesis	negative regulation of catalytic activity
M16.49											Inflammation	negative regulation of cell proliferation
M12.2											Monocytes	defense response
M13.11											TBD	response to calcium ion
M15.127											Interferon	immune response
M15.58											Monocytes	molting cycle process
M16.16											TBD	negative regulation of catalytic activity
M16.27											TBD	hemopoiesis
M16.37											TBD	regulation of cellular localization
M14.50											Inflammation	inflammatory response
M16.80											Cytokines/chemokines	epidermal growth factor receptor signaling pathway
M16.67											TBD	immune response
M16.44											Protein synthesis	immune response
M16.1											TBD	regulation of leukocyte migration
M15.66											TBD	retinoic acid metabolic process
M16.102											TBD	protein kinase cascade
M13.12											Inflammation	response to wounding
M13.16											Cytokines/chemokines	glucan catabolic process
M15.26											Neutrophils	leukocyte activation
M13.22											Neutrophils	response to bacterium
M15.37											Inflammation	apoptosis
M15.84											Cytokines/chemokines	protein kinase cascade
M12.10											Inflammation	regulation of cell morphogenesis
M15.109											Inflammation	inflammatory response
M16.82											Gene transcription	response to organic substance
M9.2											Erythrocytes	erythrocyte differentiation
M12.11											Erythrocytes	hexose metabolic process
M13.30											Erythrocytes	oxygen transport
M10.4											Neutrophil activation	defense response to bacterium
M16.96											Erythrocytes	defense response
M16.11											Protein synthesis	intracellular transport
M16.3											T cells	lymphocyte activation
M16.8											TBD	protein transport
M15.6											Cell cycle	modification-dependent macromolecule catabolic proce
M16.30											Complement	immune response

* Cells in shades of red are upregulated for the condition listed first, and shades of green if downregulated for the condition listed first.

e. Misclassified Sample Analysis:

As part of our analysis, we also examined whether there were any samples that proved to be particularly difficult to classify within our data. For the misclassified sample analysis, we examined only a fraction of our 36 configurations. Specifically, we examined the following 6 configurations: (LR + DE Feature Selection) x (Intersection/Union) x (2.5/3.0/3.5 Threshold). If a given sample was misclassified across all feature sizes in each of the 6 configurations, it was labeled as frequently misclassified. For example, if the logistic regression algorithm could never correctly classify the sample, regardless of feature size, and how the features were selected and filtered, then it was labeled as frequently misclassified. Table S30 summarizes the frequently misclassified samples found in our dataset.

Table S33: Frequently misclassified samples in each dataset.

Dataset	Frequently Misclassified Samples
LV 2-Way	None.
LV 3-Way	Two AC samples. Both misclassified as AH.
LV 5-Way	Three AC samples. Two misclassified as AH, one as NF.
PBMC 5-Way	3 AC, 1 NF, and 1 HP samples. All AC samples were primarily misclassified as AH. The NF sample was misclassified as AC. The HP sample was mostly misclassified as CT.

While the clinical data was lacking for most liver samples, the clinical data was available for all our PBMC samples. Therefore, we were able to examine whether the frequently misclassified PBMC samples were unusual in any way, based upon clinical parameters. Specifically, we examined the BMI, MELD, and DF scores. All of the frequently misclassified AC samples were notable for having some of the highest MELD and DF scores for their condition. This suggests

that severity may play a role in the way that the AC and AH conditions were being distinguished within the PBMC 5-Way dataset. The frequently misclassified NF and HP samples did not possess any unusual or outlying clinical parameters. Therefore, we could only speculate as to the reasons behind their frequent misclassification.

f. AH PBMC-LV Analysis:

Both liver and PBMC samples were collected from 19 alcohol-associated hepatitis (AH) participants. We performed differential expression analysis of 19 AH liver samples against 8 CT liver samples, and of 19 AH PBMC samples against 20 CT PBMC samples. We filtered the results using the following cutoffs: FPKM > 1, Q-Value < 0.05, and log2(FC) > 1. We then identified genes that were similarly upregulated and downregulated within both tissues as compared to CT samples from the same tissue type. As shown in Table S34, there were 37 genes that were upregulated in AH compared to CT within both tissues, and 3 genes that were downregulated in AH compared to CT within both tissues.

Table S34: Genes that were similarly upregulated and downregulated within both PBMC and LV tissues for AH vs CT comparison.

Downregulated	IFITM1, IGFBP4, MFAP3L.
Upregulated	ADAM9, AIF1, ANXA3, APOBEC3A, BLVRA, C3AR1, CPVL, CSF2RA, CTSS, FCN1, FGR, IFI44L, LGALS3, LGALS9, LILRB4, LY96, MILR1, MMP14, MNDA, MS4A4A, NCF1, NCF2, OSCAR, PECAM1, PILRA, PTAFR, SECTM1, SIRPB1, SLC11A1, SLC7A7, SNCA, ST14, TESC, TIMP2, TNFRSF21, TNFSF13B, VCAN.

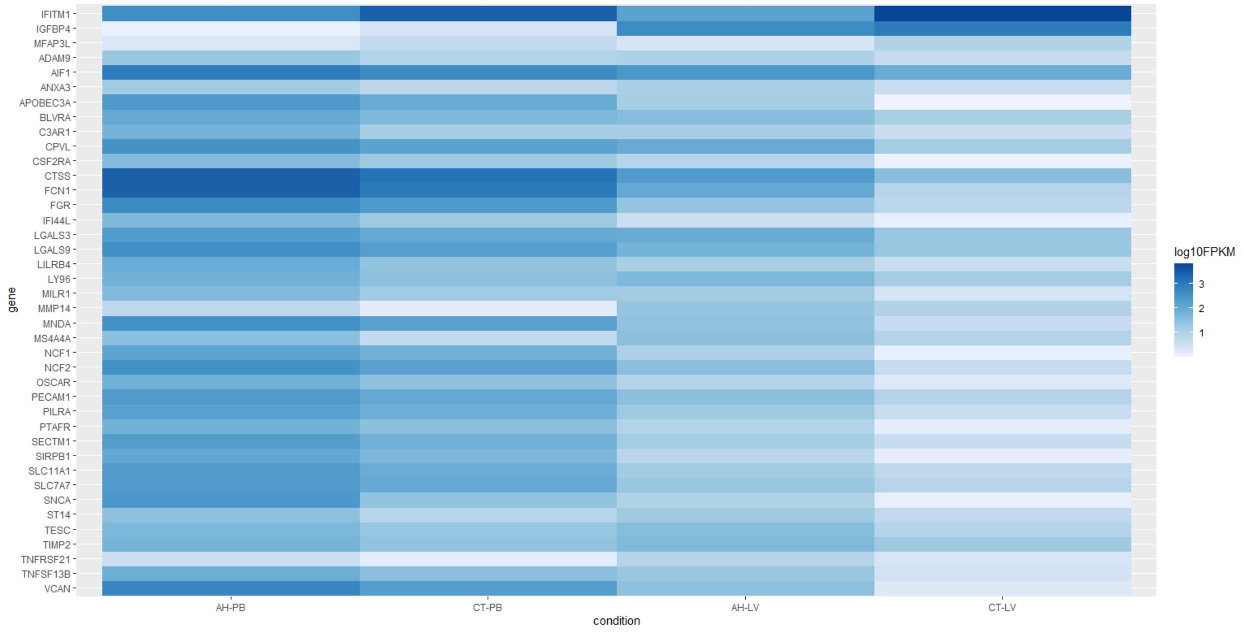

Fig. S12: Heatmap of genes that were similarly upregulated and downregulated within both PBMC and LV tissues for AH vs CT comparison.

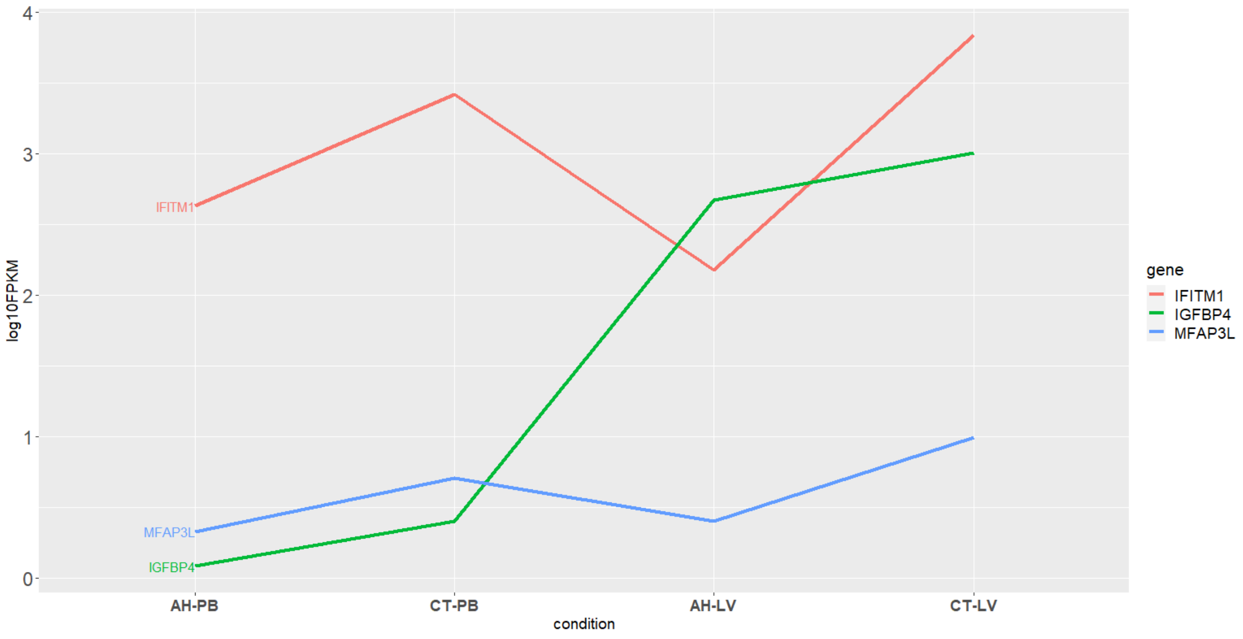

Fig. S13: Line plot of 3 genes that were downregulated in AH vs. CT, within both PBMC and liver tissues.

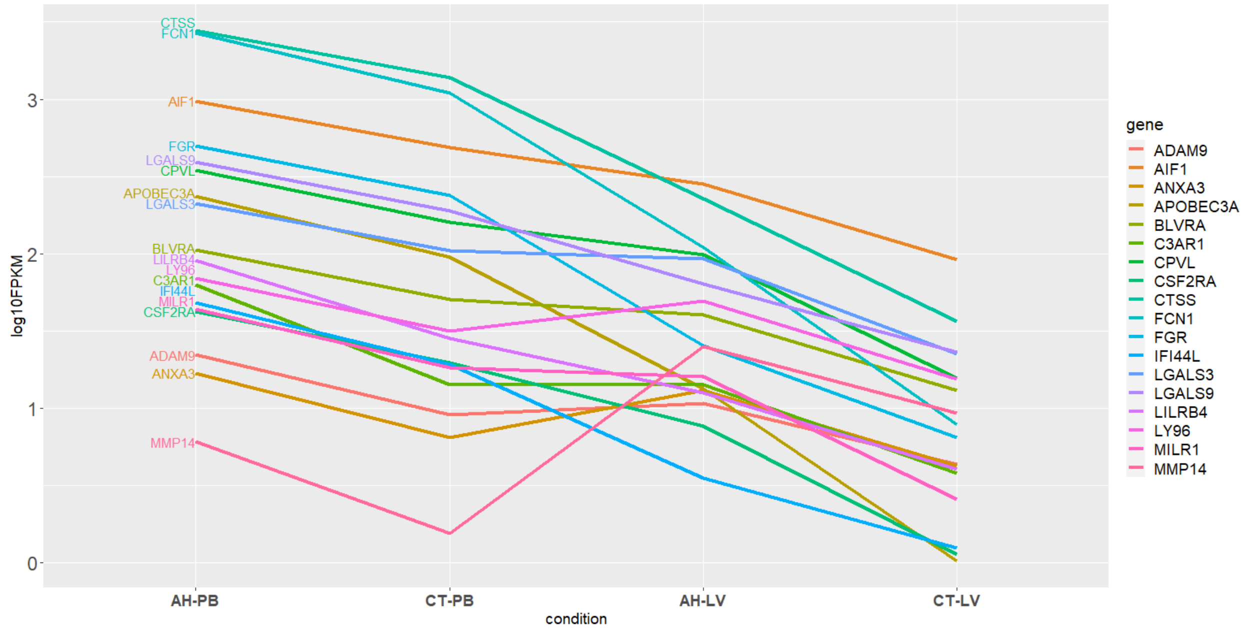

Fig. S14a: Line plot of 18 genes that were upregulated in AH vs. CT within both PBMC and liver tissues.

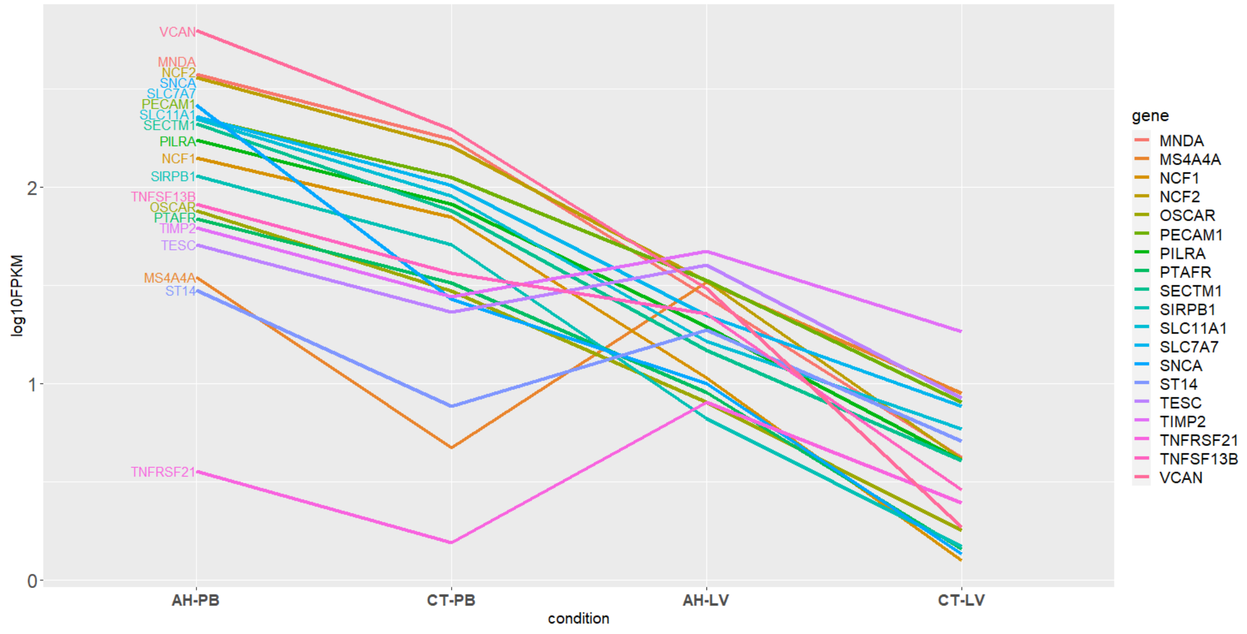

Fig. S14b: Line plot of remaining 19 genes that were upregulated in AH vs. CT within both PBMC and liver tissues.

The 40 genes that were similarly up or down regulated in both tissues are visualized by a heatmap (Fig. S12) and line plots (Figs. S13 and S14). Fig. S14 was split into two line plots to improve the readability of the individual lines. Additionally, we examined which of these 40 genes were also present in PBMC and LV 5-Way best gene sets, and found that there were 3 genes that matched exactly to our best gene sets: ANXA3, IFITM1, and IFI44L. There were also several genes belonging to the same gene families within both tissues (e.g., matrix metalloproteinase: MMP7, MMP8, MMP14; iron homeostasis: SLC25A37, SLC11A1; and Tumor Necrosis Factor: TNFS10, TNFRSF21, TNFSF13B). These genes are present in several of the key pathways that are altered during alcohol-associated hepatitis. Because these genes show similar expression directionality within both liver tissue and PBMCs, they may potentially serve as effective biomarkers for AH.

Supplementary references

1. Massey V, Parrish A, Argemi J, Moreno M, Mello A, García-Rocha M, et al. Integrated Multiomics Reveals Glucose Use Reprogramming and Identifies a Novel Hexokinase in Alcoholic Hepatitis. Gastroenterology. 2021;160(5):1725-1740.

2. Kim D, Pertea G, Trapnell C, Pimentel H, Kelley DR, Salzberg SL. TopHat2: accurate alignment of transcriptomes in the presence of insertions, deletions and gene fusions. Genome Biology. 2013;14.

3. Langmead B, Salzberg SL. Fast gapped-read alignment with Bowtie 2. Nature Methods 2012;9:357-U354.

4. Kim D, Paggi JM, Park C, Bennett C, Salzberg SL. Graph-based genome alignment and genotyping with HISAT2 and HISAT-genotype. Nature Biotechnology. 2019;37:907-915.

5. Dobin A, Davis C, Schlesinger F, Drenkow J, Zaleski C, Jha S, et al. STAR: ultrafast universal RNA-seq aligner. Bioinformatics. 2013;29(1):15-21.

6. Kampf C, Mardinoglu A, Fagerberg L, Hallstrom BM, Edlund K, Lundberg E, et al. The human liver-specific proteome defined by transcriptomics and antibody-based profiling. Faseb Journal 2014;28:2901-2914.

7. Bolger AM, Lohse M, Usadel B. Trimmomatic: a flexible trimmer for Illumina sequence data. Bioinformatics 2014;30:2114-2120.

8. Trapnell C, Roberts A, Goff L, Pertea G, Kim D, Kelley DR, et al. Differential gene and transcript expression analysis of RNA-seq experiments with TopHat and Cufflinks. Nature Protocols 2012;7:562-578.

9. Hart SN, Therneau TM, Zhang YJ, Poland GA, Kocher JP. Calculating Sample Size Estimates for RNA Sequencing Data. Journal of Computational Biology 2013;20:970-978.

10. Chen EY, Tan CM, Kou Y, Duan QN, Wang ZC, Meirelles GV, et al. Enrichr: interactive and collaborative HTML5 gene list enrichment analysis tool. Bmc Bioinformatics 2013;14.

11. Krämer A, Green J, Pollard J, Tugendreich S. Causal analysis approaches in Ingenuity Pathway Analysis. Bioinformatics. 2014;30(4):523–30.

12. Mootha V, Lindgren C, Eriksson KF, Subramanian A, Sihag S, Lehar J, et al. PGC-1α-responsive genes involved in oxidative phosphorylation are coordinately downregulated in human diabetes. Nature Genetics. 2003;34:267–273.

13. Rinchai D, Roelands J, Toufiq M, Hendrickx W, Altman MC, Bedognetti D, et al. BloodGen3Module: blood transcriptional module repertoire analysis and visualization using R. Bioinformatics. 2021;37(16):2382–2389.

14. Li S, Rouphael N, Duraisingham S, Romero-Steiner S, Presnell S, Davis C, et al. Molecular signatures of antibody responses derived from a systems biology study of five human vaccines. Nature Immunology. 2014;15:195–204.

15. Sharma S, Baweja S, Maras JS, Shasthry SM, Moreau R, Sarin SK. Differential blood transcriptome modules predict response to corticosteroid therapy in alcoholic hepatitis. JHEP Reports. 2021;3(3).
