# Supplementary material for: Differentiating between liver diseases by applying multiclass machine learning approaches to transcriptomics of liver tissue or blood-based samples
Source: JHEP Rep. 2022 Aug 18;4(10):100560. doi: 10.1016/j.jhepr.2022.100560 (PMC9472076; doi:10.1016/j.jhepr.2022.100560)
Supplement: Multimedia component 2 [file mmc2.pdf]

JHEP Reports

CTAT methods

Tables for a “Complete, Transparent, Accurate and Timely account” (CTAT) are now mandatory for all revised submissions. The aim is to enhance the reproducibility of methods.

- Only include the parts relevant to your study
- Refer to the CTAT in the main text as ‘Supplementary CTAT Table’
- Do not add subheadings
- Add as many rows as needed to include all information
- Only include one item per row

If the CTAT form is not relevant to your study, please outline the reasons why:

1.1 Antibodies

Name	Citation	Supplier	Cat no.	Clone no.
NA				

1.2 Cell lines

Name	Citation	Supplier	Cat no.	Passage no.	Authentication test method
NA					

1.3 Organisms

Name	Citation	Supplier	Strain	Sex	Age	Overall n number
NA						

1.4 Sequence based reagents

Name	Sequence	Supplier
NA		

1.5 Biological samples

Description	Source	Identifier
PBMC	Southern California Alcoholic Hepatitis Consortium (SCAHC)	NA
Liver Tissue	SCAHC and University of Minnesota Liver Tissue Cell Distribution System (LTCDS)	NA

1.6 Deposited data

Name of repository	Identifier	Link
RNAseq – dbGAP controlled access	In process upon acceptance	In process upon acceptance

1.7 Software

Software name	Manufacturer	Version
Cuffdiff (Cufflinks)	Open source	2.2.1
Enrichr	Open source	NA
Ingenuity Pathway Analysis (IPA)	Qiagen	Winter 2021
GSEA, GSEAPreranked	Open source	4.2.3
BloodGen3Module	Open source	1.4.0
Python	Open source	3.7+
RNASeqPower	Open source	1.34.0
Scikit-learn	Open source	0.24.2+
STAR	Open source	2.6.0

1.8 Other (*e.g.* drugs, proteins, vectors etc.)

NA		

1.9 Please provide the details of the corresponding methods author for the manuscript:

Trina M. Norden-Krichmar
Department of Epidemiology and Biostatistics
University of California
Irvine, CA 92697 USA

Phone number: +1 949-824-8802
Fax number: None
Email: tnordenk@uci.edu

2.0 Please confirm for randomised controlled trials all versions of the clinical protocol are included in the submission. These will be published online as supplementary information.

NA
